# Development of a high affinity Anticalin^®^ directed against human CD98hc for theranostic applications

**DOI:** 10.7150/thno.38968

**Published:** 2020-01-12

**Authors:** Friedrich-Christian Deuschle, Volker Morath, André Schiefner, Corinna Brandt, Simone Ballke, Sybille Reder, Katja Steiger, Markus Schwaiger, Wolfgang Weber, Arne Skerra

**Affiliations:** 1 Lehrstuhl für Biologische Chemie, Technische Universität München, 85354 Freising, Germany; 2 Department of Nuclear Medicine, Klinikum rechts der Isar, School of Medicine, Technical University of Munich, 81675 München, Germany; 3 Institute of Pathology, Klinikum rechts der Isar, School of Medicine, Technical University of Munich, 81675 München, Germany

**Keywords:** cancer theranostics, CD98hc, lipocalin, PASylation, protein engineering, tumor targeting

## Abstract

Enhanced amino acid supply and dysregulated integrin signaling constitute two hallmarks of cancer and are pivotal for metastatic transformation of cells. In line with its function at the crossroads of both processes, overexpression of CD98hc is clinically observed in various cancer malignancies, thus rendering it a promising tumor target.

**Methods**: We describe the development of Anticalin proteins based on the lipocalin 2 (Lcn2) scaffold against the human CD98hc ectodomain (hCD98hcED) using directed evolution and protein design. X-ray structural analysis was performed to identify the epitope recognized by the lead Anticalin candidate. The Anticalin - with a tuned plasma half-life using PASylation^®^ technology - was labeled with ^89^Zr and investigated by positron emission tomography (PET) of CD98-positive tumor xenograft mice.

**Results**: The Anticalin P3D11 binds CD98hc with picomolar affinity and recognizes a protruding loop structure surrounded by several glycosylation sites within the solvent exposed membrane-distal part of the hCD98hcED.* In vitro* studies revealed specific binding activity of the Anticalin towards various CD98hc-expressing human tumor cell lines, suggesting broader applicability in cancer research. PET/CT imaging of mice bearing human prostate carcinoma xenografts using the optimized and ^89^Zr-labeled Anticalin demonstrated strong and specific tracer accumulation (8.6 ± 1.1 %ID/g) as well as a favorable tumor-to-blood ratio of 11.8.

**Conclusion**: Our findings provide a first proof of concept to exploit CD98hc for non-invasive biomedical imaging. The novel Anticalin-based αhCD98hc radiopharmaceutical constitutes a promising tool for preclinical and, potentially, clinical applications in oncology.

## Introduction

Elevated amino acid transport, altered cell adhesion and migration as well as adhesive signaling constitute crucial factors for cancer development and growth and play a critical role for metastatic transformation of cells. The cluster of differentiation 98 heavy chain (CD98hc, also known as 4F2hc, SLC3A2 or FRP-1) is involved in both pathophysiological processes and therefore represents an interesting therapeutic target 1. In the Human Protein Atlas 2 CD98hc (gene name / entry: SLC3A2) shows broad expression in healthy tissues as well as cancers, predominantly as an unfavorable prognostic marker.

**Figure 1:**
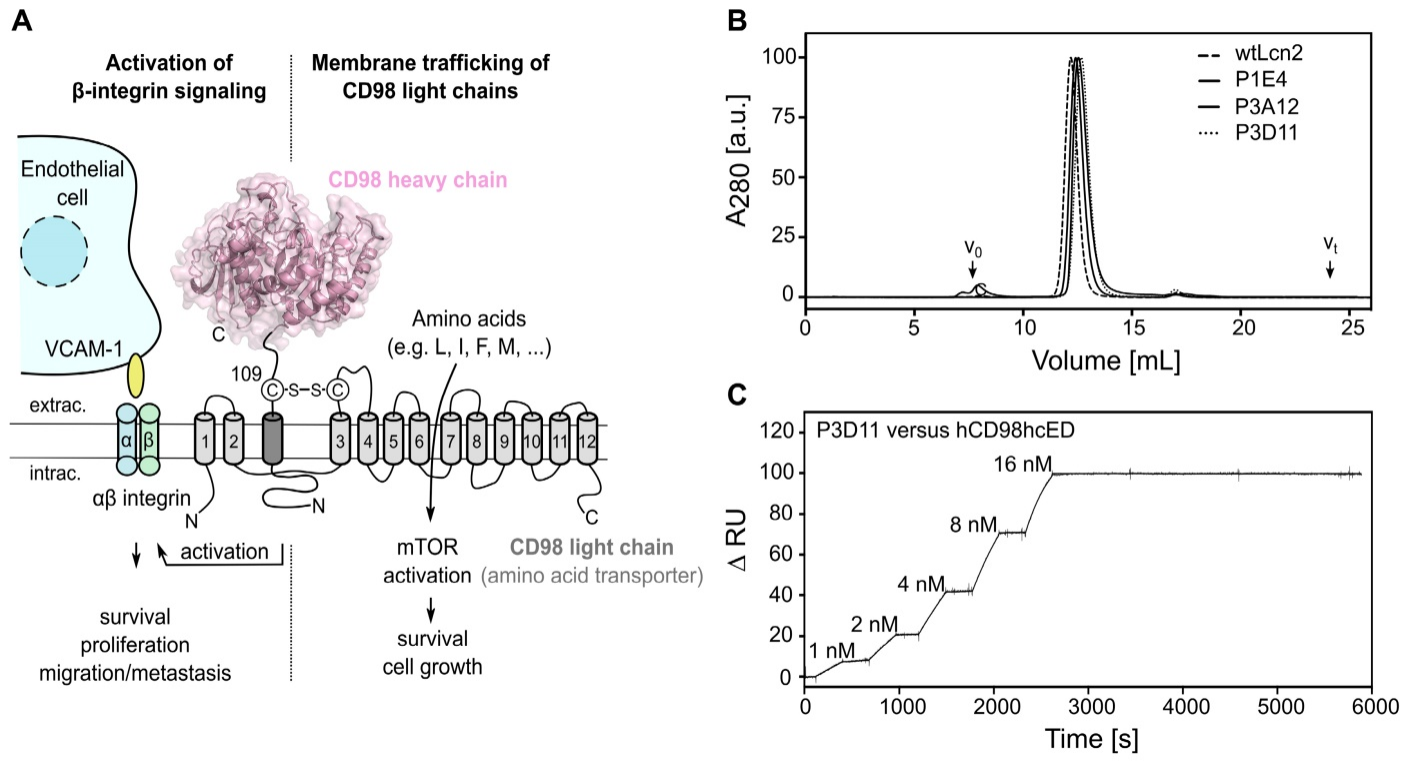
** Phage display selection of lipocalin variants with affinity towards the ectodomain of hCD98hc.** (A) Graphical depiction of CD98hc linked to a CD98 light chain via a disulfide bridge, illustrating the two main biochemical functions: amino acid transport and integrin signaling. The structure of the CD98hc ectodomain, which was employed as a recombinant protein for phage display selection of Anticalins is highlighted in pink. (B) Analytical SEC of the CD98hc-specific lipocalin variants P1E4, P3A12 and P3D11 that were selected via phage display, in comparison with wtLcn2 (all purified as soluble proteins expressed in *E. coli*), revealing monomeric protein preparations (minor aggregate contaminations elute at the void volume, V_0_). (C) SPR real-time binding analysis (single cycle) of the high-affinity variant P3D11 *versus* hCD98hcED, demonstrating a picomolar dissociation constant (see **Table 1**).

Structurally, CD98hc is a type-II-transmembrane glycoprotein which is disulfide-linked to one of six known multi-pass CD98 light chains (CD98lc), all of which are permease-type amino acid transporters with different specificities 3, namely Lat-1 4, Lat-2 5, y+Lat-1 6, y+Lat-2 7, asc-1 8 and xCT 9. CD98hc has two major biochemical functions (**Figure 1A**). First, it acts as a molecular chaperone for membrane trafficking, stabilization and proper function of the CD98lc 10, 11, thus boosting the transport of amino acids and contributing to cell survival and growth. Of note, beside serving as building blocks for biosynthesis, the amino acids transported by the CD98 complex and their metabolites/derivatives can exert further cellular effects, as described for L-leucine (mTOR1 pathway activation 12) and L-cystine (regulation of redox homeostasis 13), which are transported by the CD98hc/Lat-1 and CD98hc/xCT heterodimers, respectively 14. Second, CD98hc directly contributes to intracellular integrin signaling and is involved in integrin-mediated cell to cell interactions via cellular adhesion molecules, such as VCAM-1, thereby influencing cell growth, survival, spreading and migration 15-19.

In line with its important role in cellular metabolism and adhesive signaling, overexpression of CD98hc has been detected in solid and hematological human malignancies, including colorectal cancer 20, non-small cell lung cancer (NSCLC) 21, 22, triple-negative breast cancer 23, 24, metastatic prostate cancer 25 as well as lymphoma 26 and leukemia 15, 22. Recent studies have indicated that abundant expression of CD98hc in cancer is associated with poor clinical prognosis 20, 21, treatment response 27, 28 as well as overall survival 24, 29, 30. Furthermore, high CD98hc expression is linked to a progressive and metastatic phenotype in several human neoplasms; consequently, assessment of CD98hc expression should aid the biological characterization of malignancies 31-34.

Likewise, elevated expression of the covalently associated CD98lc components Lat-1 35, 36, Lat-2 37 and xCT 38, 39 is also observed in human cancers, with Lat-1 constituting the most abundant tumor marker. In fact, cancers from several tissues highly express both CD98hc and Lat-1, suggesting that this receptor heterodimer bears particularly high oncogenic potential. Interestingly, in some cancers a cooperative overexpression of CD98hc and Lat-1, contrasting with Lat-1 alone, has emerged as an independent factor of poor prognosis for patients 24, 40, 41.

Therefore, the specific recognition and/or targeting of CD98hc by appropriate protein reagents offers potential for cancer theranostics. To date, the most advanced protein drug candidate is the humanized αhCD98hc monoclonal antibody (mAb) IGN523, which has shown robust preclinical anti-tumor activity in patient-derived lymphoma as well as NSCLC xenograft tumor models and a favorable safety profile in a phase I clinical study 22, 42. However, protein reagents suitable for the non-invasive diagnostic imaging of CD98hc have not been described to date. On the other hand, certain small molecules are available to target Lat-1, demonstrating that this CD98lc subtype is also a promising target for tumor imaging and therapy. These include inhibitor drugs with proven preclinical antitumor activity in different cancer types 43 and the radiolabeled amino acid analogues [^18^F]-fluoroethyl-L-tyrosine (FET) and trans-1-amino-3-[^18^F]-fluorocyclobutanecarboxylic acid (FACBC). While FET is widely used in Europe for PET imaging of brain tumors 44, the leucine analog FACBC has recently been approved in the United States and Europe for PET imaging of recurrent prostate cancer 45. Apart from that, a ^89^Zr-labeled αLat-1 mAb has shown tumor uptake in a colorectal cancer xenograft model 46. However, mAbs suffer from drawbacks as imaging reagents, due to their poor tissue penetration and unfavorable pharmacokinetics, which causes low imaging contrast 47. Furthermore, the direct targeting of CD98hc would offer additional information on pathophysiological processes beyond the amino acid transport catalyzed by the associated CD98lc.

Here, we describe the development and preclinical investigation of a hCD98hc-specific Anticalin. Anticalins constitute an emerging class of artificial binding proteins obtained by combinatorial design based on the compact and robust human lipocalin scaffold 48. Due to their human origin, these proteins have low immunogenic potential and Anticalins with various target specificities have demonstrated safety in clinical trials 49. Anticalins are particularly well suited for applications in cancer therapy and diagnostics due to their small size and good tissue penetration, tunable pharmacokinetics (e.g. via PEGylation or PASylation 47, 50), the possibility for site-specific labeling (with PET/SPECT radioisotopes or optical imaging probes), drug conjugation (toxins) and flexible formatting options as fusion proteins 49.

## Materials and Methods

### Phage display selection and production of hCD98hcED-specific lipocalin variants

Selection of hCD98hc-specific lipocalin variants was performed via filamentous phagemid display from a combinatorial library comprising 1×10^10^ variants based on Lcn2 randomized at 20 positions within the structurally variable loops 51. Biotinylated recombinant hCD98hcED (cf. Supplementary Information) was immobilized on streptavidin- or NeutrAvidin-coated paramagnetic beads (Sigma-Aldrich, Munich, Germany and Thermo Fisher Scientific, Waltham, MA, respectively), then incubated with the phagemid library (starting titer 1×10^12^) and, following extensive washing, bound phagemids were eluted under denaturing conditions using 4 M urea. After five consecutive cycles, pooled phasmid DNA from the enriched library was prepared and subcloned on pNGAL98 in order to perform high-throughput ELISA screening from microcultures expressing the soluble lipocalin variants with a C-terminal *Strep*-tag II, as previously described 51. Briefly, the periplasmic extract was prepared in a microtiter plate and transferred to a 96-well MaxiSorp plate (Thermo Fisher Scientific) that had been coated with 10 µg/mL *Strep*MAB-Immo (IBA, Göttingen, Germany). After 1 h incubation and washing steps, bound lipocalin variants were incubated with the biotinylated hCD98hcED, followed by detection of lipocalin•target complexes using ExtrAvidin/alkaline phosphatase (AP) conjugate (Sigma-Aldrich). Signals were developed with 0.5 mg/mL p-nitrophenyl phosphate in AP buffer (0.1 M NaCl, 5 mM MgCl_2_, 0.1 M Tris/HCl, pH 8.8) and absorbance was measured at 405 nm with a Synergy 2 photometer (BioTek Instruments, Winooski, VT). For clones showing significant binding towards hCD98hcED the expression cassette on the phasmid was sequenced, and the soluble lipocalin variant was subsequently produced at preparative scale in *E. coli* JM83 with a C-terminal His_6_-tag using the plasmid pNGAL118 51. After periplasmic protein extraction, the recombinant protein was purified by immobilized metal ion affinity chromatography (IMAC) using a Ni(II)-charged HisTrap HP column (GE Healthcare, Munich, Germany) and finally subjected to size-exclusion chromatography (SEC) in PBS (4 mM KH_2_PO_4_, 160 mM Na_2_HPO_4_, 115 mM NaCl pH 7.4) on a 24 mL Superdex 75 10/300 GL column (GE Healthcare). Plasmid construction and production of PASylated lipocalin variants D11vs-PAS200, D11vs-PAS200-Cys and wild type (wt) Lcn2-PAS200-Cys was performed according to published procedures 52 and followed by purification via IMAC and SEC essentially as described above.

### Biomolecular interaction analysis

Real time surface plasmon resonance (SPR) spectroscopy was performed on a BIAcore 2000 system (BIAcore, Uppsala, Sweden) at 25 °C using HBS-T (20 mM Hepes/NaOH pH 7.5, 150 mM NaCl, 0.005% (v/v) Tween 20) as running buffer. The purified biotinylated m/hCD98hcED produced in *E. coli* or in MEXi-293E cells (3 µg/mL protein solution in HBS-T; see Supplementary Information) was immobilized (∆RU ≈ 225) on a streptavidin-functionalized sensorchip using the Biotin CAPture kit (GE Healthcare). Single cycle kinetic experiments were performed using five consecutive injections from a 1:2 dilution series of the purified lipocalin variant at a flow rate of 25 µL/min, each with 288 s contact time, and a long 3500 s dissociation time after the fifth injection. For conventional multi cycle kinetic experiments, association and dissociation were recorded for eight samples from a consecutive 1:2 dilution series of the purified lipocalin variant. Rate constants of association and dissociation were calculated from reference-corrected sensorgrams by fitting to a global 1:1 Langmuir binding model using BIAevaluation software (BIAcore). The equilibrium dissociation constant (K_D_ value) was calculated as k_off_/k_on_. To test competitive binding of the lipocalin variants P3D11, P3A12 and P1E4 to immobilized hCD98hcEDg (cf. Supplementary Information), 100 nM of P3D11 was first injected, followed by an injection of either 100 nM P3A12 or 100 nM P1E4. Injections of HBS-T served as negative control (cf. **Figure S2E**).

### Error-prone library construction and selection via bacterial surface display

Stability and affinity engineering of the lipocalin variant P3D11 was accomplished by directed evolution using bacterial surface display (BSD) as previously described 52-54. Briefly, error-prone PCR was executed using the GeneMorph II random mutagenesis kit (Agilent Technologies, Santa Clara, CA) with 10 pg of the preamplified coding region for the lipocalin variant P3D11 used as DNA template. After digest of the PCR product with *Bst*XI, the randomized DNA fragment was ligated with the backbone of pNGAL146 and used for electrotransformation of *E. coli* JK321 55, yielding 5×10^9^ transformants, which were plated on LB agar medium supplemented with 100 µg/mL ampicillin (LB/Amp). The bacterial lawn was scraped from the plate(s), resuspended in 50 mL liquid LB/Amp medium to reach an initial OD_550_ = 0.15 and incubated at 37 °C. Gene expression was induced at OD_550_ = 0.5 by addition of 10 ng/mL anhydrotetracycline (aTc) for 2.5 h. Approximately 2×10^8^ cells were sedimented by centrifugation and resuspended in PBS in the presence of biotinylated hCD98hcED at different concentrations (cycles 1 and 2: 100 nM; cycles 3 and 4: 10 nM; cycles 5 and 6: 1 nM) followed by incubation for 1 h at 4 °C. After centrifugation and one washing step with PBS, the bacteria were incubated with 25 µg/mL streptavidin/phycoerythrin (PE) conjugate (Biolegend, San Diego, CA) and 3 µM Fab A3C5-DY634 conjugate 56 in PBS for 30 min, followed by a last washing step in PBS. Bacteria were sorted on a FACSAria IIu instrument (BD Bioscience, Heidelberg, Germany) using a 488 nm LASER diode in combination with a 585/42 band pass filter or a 633 nm HeNe LASER with a 660/20 band pass filter for excitation/detection of PE and DY634 fluorescence, respectively. Sorted bacteria were plated on LB/Amp agar, incubated at 37 °C for 12 h and then subjected to a new sorting cycle. After cycle no. 6, single clone analysis was performed by cytofluorometric analysis of individually cultured colonies. FACS data were processed using FlowJo ver.10 software (FlowJo, Ashland, OR).

### Flow cytofluorometric of cell lines

The human cancer cell lines Ramos 57, Raji 58, SU-DHL-4 59, PC-3 60, DU-145 61 and Caco-2 62 (from local collections) were cultivated in Roswell Park Memorial Institute (RPMI) 1640 medium (containing 2.0 g/L NaHCO_3_ and stable L-glutamine; Biochrom, Berlin, Germany), supplemented with 10% (v/v) fetal bovine serum (FBS; PAA Laboratories, Pasching, Austria) at 37 °C under humidified 5% CO_2_ atmosphere. The HEK293T cell line and a corresponding CD98hc knock-out clone 63 were maintained under cultivation conditions as published 64. Adherent cells (PC-3, DU-145, Caco-2 and HEK293T-based cell lines) were washed with PBS (w/o Ca^2+^ and Mg^2+^; Biochrom) and detached using PBS (w/o Ca^2+^ and Mg^2+^) supplemented with 0.5 mM EDTA for 10 min at 37 °C. Cells growing in suspension (Ramos, Raji and SU-DHL-4) were directly washed with PBS (w/o Ca^2+^ and Mg^2+^). For each measurement, 250,000 viable cells were resuspended in 250 µL FACS buffer (PBS + 10% (v/v) FBS) supplemented with 1 µM D11vs-PAS200-Cy5.5 (cf. Supplementary Information) and incubated for 1 h at 4 °C. Competition experiments were performed by adding a 10-fold molar concentration of either unlabeled D11vs-PAS200 or the soluble hCD98hcEDg. The Sulfo-Cy5.5 conjugate of recombinant wtLcn2-PAS200 was used as a negative control in some experiments (see text). After incubation, cells were centrifuged and washed three times in PBS and finally resuspended in 250 µL FACS buffer. Flow cytofluorometric experiments were performed on a FACSAria IIu instrument using an excitation wavelength of 650 nm and an emission band-path filter of 780/60 nm.

### Immunofluorescence microscopy

PC-3 and Caco-2 cells were cultivated at 37 °C on poly-D-lysine (PDL) coated Lab-Tek II chamber slides (Thermo Fisher Scientific) in RPMI 1640 medium containing 2.0 g/L NaHCO_3_ and stable L-glutamine, supplemented with 10% (v/v) FBS, until 60-70% confluence was reached. After three times washing with PBS, the cells were incubated with 1 µM D11vs-PAS200-Cy5.5 for 1 h at 37 °C, followed by another three washing steps. Ramos cells, on the other hand, were grown in suspension and stained, after centrifugation and resuspension, in a similar manner, followed by transfer to a PDL coated Lab-Tek II chamber slide. For control experiments, the D11vs-PAS200-Cy5.5 conjugate was either premixed with a 10-fold molar concentration of unlabeled D11vs-PAS200 or wtLcn2-PAS200-Cy5.5 was applied. Cells were fixed and counterstained by 5 min incubation with ice-cold methanol containing 1 µg/mL 4',6-diamidino-2-phenylindole (DAPI; Sigma-Aldrich). Digital fluorescence images were recorded on an Axiovert 40 CFL microscope equipped with an AxioCam MRm camera (Carl Zeiss Microscopy, Jena, Germany) using 365/12 nm (DAPI) and 716/40 nm (Cy5.5) band-pass filters.

### Circular dichroism (CD) spectroscopy and thermal denaturation

CD spectra and thermal unfolding of purified lipocalin variants were measured using a J-810 spectropolarimeter (Jasco, Pfungstadt, Germany) equipped with a PT-423S Peltier element and controlled by Spectra manager software (ver. 1.53.05). Proteins were dialyzed against 20 mM KP_i_ pH 7.5, 50 mM K_2_SO_4_ and applied at a concentration of 1 µM. In order to identify the wavelength with maximum change in CD signal for thermal denaturation studies, spectra were first recorded with a 1 mm path length quartz cuvette (Hellma, Müllheim, Germany) from 190 to 250 nM both at 20 °C and 90 °C. Thermal unfolding of the lipocalin variants was then measured at a wavelength of 214 nm by heating from 20 °C to 90 °C at a rate of 60 K/h. Data were fitted to an equation for a one-step unfolding transition, and the melting temperature (T_m_), enthalpy of unfolding (∆H_m_) and Gibb's free energy of unfolding (∆G^0^) at ambient temperature were calculated as previously described 65.

### *In vivo* xenograft studies

Animal experiments were conducted with permission from the District Government of Upper Bavaria (application no.: 55.2-1-54-2532-216-15). CB17-SCID mice at an age of 6 weeks (♂ and ♀) were purchased from Charles River Laboratories (Wilmington, MA) and held under specific pathogen free (SPF) conditions. Mice were injected subcutaneously above the right shoulder with 100 µL RPMI 1640 medium (without supplements) containing 6×10^6^ PC-3 or Ramos cells. On day 12 post injection (p.i.) the tumor had reached a diameter of ~0.5 cm and the mice were used for *in vivo* studies.

### ^89^Zr Positron Emission Tomography (PET)

For ^89^Zr-labeling of Dfo-conjugated 66 D11vs-PAS200 (cf. Supplementary Information), 250 µg of the protein conjugate solution which had been dialyzed against 250 mM Na-acetate pH 5.5 was incubated with 200 µL 0.5 M Hepes/NaOH pH 7.0, 50 µL 0.5 M gentisic acid and 111 MBq ^89^Zr(IV), obtained in 1 M oxalic acid (Perkin Elmer, Waltham, MA) and neutralized with 3 M Na_2_CO_3_. After 1 h incubation at 37 °C, radiolabeling was confirmed by radio thin layer chromatography (radio-TLC) on chromatography paper strips (TLC-SG; Agilent Technologies) using Na-citrate pH 5.0 as mobile phase. The radio-labeled protein tracer was separated from reagents by gel filtration using a PD-10 column (GE Healthcare) equilibrated with 0.9% NaCl solution (B. Braun, Melsungen, Germany). Radiochemical purity of the protein tracer was confirmed by radio-TLC prior to its use for injections.

For the biodistribution study, mice were injected with the protein tracer via the tail vein at a dose of 3.96 ± 0.12 MBq. For blocking experiments, a 100-fold or 250-fold molar equivalent of unlabeled D11vs-PAS200 was injected 2 h prior to the tracer injection. Static PET/CT imaging was performed at indicated time points under isoflurane anesthesia in an Inveon PET/CT small-animal scanner (Siemens Medical Solutions, Erlangen, Germany) with 20 min PET and 5 min CT signal acquisition. Data were analyzed with the Inveon Research Workplace software (Siemens Medical Solutions) and reconstructed using the 0.8 mm high resolution OSEM-3D algorithm. Quantification of regions of interest (ROI) was performed by threshold-based image segmentation with a lower threshold at 50% of the hottest voxel for *in vivo* PET and a threshold of 10 kBq/mL for *ex vivo* PET images.

### Biodistribution analysis and tissue autoradiography

Tumor, blood and organs were dissected from the sacrificed animals at the end of the experiment in order to determine weight and quantify radioactivity using a 2480 Wizard^2^ automatic gamma counter (Perkin Elmer). 1% of the injected protein tracer dose was applied as reference. Uptake values were calculated as %ID/g and corrected for radioactive decay from the time point of injection.

After radioactivity measurement, the tumor was transversally bisected and used for immunohistochemistry and autoradiography analysis. For autoradiography, the tumor was embedded in Tissue-Tek O.C.T. (Sakura Finetek, Alphen aan den Rijn, The Netherlands) in a mold and frozen. Subsequently, 10 µm sections were cut using a CM1950 cryostat (Leica Biosystems, Nußloch, Germany) and the sections were mounted on a superfrost microscopic slide (Thermo Fisher Scientific). The slide was placed, together with a 1:2 dilution row of the radiotracer, in silicon isolators (Grace Bio-Labs, Bend, OR) on a storage phosphor screen BAS-IP super resolution film (GE Healthcare) for one week, followed by quantification using a CR 25 BIO autoradiography scanner (Dürr Medical, Bietigheim-Bissingen, Germany) and data analysis with AIDA software ver. 4.24.036 (Raytest, Straubenhardt, Germany). Pictures chosen show the sections with the highest exposure level in order to allow optimal comparison of blocked and unblocked tumor sections.

### Histology and immunohistochemistry

Tumor tissue was fixed in 10% (w/v) neutral-buffered formalin solution (Otto Fischar, Saarbrücken, Germany) for 48 h and stored at 4 °C until the radioactivity had decayed. Tissue samples were dehydrated using an automated system (ASP300S; Leica Biosystems) and subsequently embedded in paraffin. Serial 2 µm sections were prepared with a rotary microtome (HM355S; ThermoFisher Scientific) and subjected to histological and immunohistochemical analysis. Hematoxylin and eosin (H&E) staining was performed on deparaffinized sections with Eosin and Mayer's Haemalaun (Morphisto, Frankfurt am Main, Germany). Immunohistochemistry was performed using a Bond RXm system (Leica Biosystems) with primary antibodies against CD98hc (E-5; Santa Cruz Biotechnology, Dallas, TX) and CD31 (Dianova, Hamburg, Germany) using 1:750 and 1:100 dilutions, respectively. Briefly, slides were deparaffinized using deparaffinization solution (Leica Biosystems), pretreated with Epitope retrieval solution 1 (corresponding to citrate buffer, pH 6) for 30 min (CD98hc) or pretreated with Epitope retrieval solution 2 (corresponding to EDTA based buffer, pH 9) for 30 min (CD31). Bound antibody was detected with a polymer refine detection kit without post primary reagent and with an intermediate rabbit anti-rat secondary antibody (diluted 1:400; Leica Biosystems) and signals were developed with 3,3'-diaminobenzidine (DAB). Representative images were collected on an Aperio AT2 digital pathology slide scanner using ImageScope (ver.12.3) software (both from Leica Biosystems).

### Statistics

For mean comparison between two groups of animals, the Student's *t* test for unpaired data was used. *p* values ≤ 0.05 were considered statistically significant (* p ≤ 0.05; ** p ≤ 0.005). Experimental data were analyzed using Prism 6 (GraphPad Software, San Diego, CA), and standard deviations (S.D.) are indicated.

## Results

### Selection of a hCD98hcED-specific Anticalin with picomolar affinity

The soluble monomeric extracellular domain of human CD98hc (UniProt ID P08195-2, residues Glu111-Ala529; calculated mass 48.4 kDa) was produced both as unglycosylated protein in *E. coli* (hCD98hcED) and fully glycosylated in human embryonic kidney (HEK) 293E cells (hCD98hcEDg) (**Figure S1**). The four experimentally verified N-glycosylation sites 67 of the native hCD98hc, Asn264, Asn280, Asn323 and Asn405, account for 32% of the apparent mass of the recombinant hCD98hcEDg (73.1 kDa), in agreement with its elution behavior in analytical SEC and its retarded mobility in SDS-PAGE (**Figure S1 and Table S1**). This extensive glycosylation of hCD98hcED may hamper *in vitro* phage display selection of cognate Anticalins due to limited sterical accessibility of potential surface epitopes, as previously seen in the selection of Anticalins against the prostate-specific membrane antigen (PSMA) 68. To avoid the necessity of glycan removal, which requires harsher conditions to prevent incomplete deglycosylation, we used the unglycosylated ectodomain produced in *E. coli* as the initial molecular target for phage display selection. N-terminal fusion of the biotin acceptor peptide (BAP) 69 resulted in the intracellular enzymatic attachment of a single biotin group to hCD98hcED upon co-expression of the BirA ligase (verified via western blot analysis, data not shown) for both expression systems used. This modification allowed immobilization of the purified ectodomain to (strept)avidin-coated beads, or surfaces, in an orientation that resembles the *in vivo* situation for the type II membrane protein, rendering its epitopes accessible for binding of lipocalin variants during phage display selection, ELISA screening and *in vitro* binding studies.

Anticalin candidates were selected from a Lcn2 random library with a combinatorial complexity of 1×10^10^, which had served for the development of other hapten- and protein-specific Anticalins in the past 51. After six selection cycles with hCD98hcED, three independent cognate lipocalin variants were identified by ELISA screening. These were expressed as soluble proteins in *E. coli* at the shake flask scale and purified to homogeneity, resulting in monomeric proteins with apparent molecular sizes similar to recombinant wild type (wt) Lcn2 (**Figures 1B and S2A**-**B, Table S2**). To determine the rate constants of association and dissociation of the selected Anticalin candidates, SPR real-time interaction analyses were performed using both the glycosylated and unglycosylated immobilized CD98hcED. The three lipocalin variants showed equilibrium dissociation constants (K_D_) in the single-digit to sub-nanomolar range (**Figures 1C and S2C-D, Tables 1 and S3**). The lipocalin variant P3D11 revealed a particularly low K_D_ value of 150-170 pM for hCD98hcED and hCD98hcEDg, as well as a long complex half-life of 6 h, hence suggesting suitability for tumor targeting *in vivo* (**Figure 1C**). Interestingly, competitive SPR experiments indicated that all three Anticalins recognize overlapping epitopes on hCD98hcED (**Figure S2E,** shown for P3D11 and P3A12), despite considerable sequence deviation especially between the variants P1E4 and P3D11 (cf. **Figure S2A**).

### Protruding loops of hCD98hcED constitute the major epitope recognized by the engineered lipocalin

To identify the epitope region and to understand the structural mechanisms of the tight molecular interaction between P3D11 and the CD98hc ectodomain, we crystallized this lipocalin variant in complex with hCD98hcED (carrying an N-terminal *Strep*-tag II) after isolation of the 1:1 complex via SEC (**Figure S3**). Crystals belonged to the space group C2, with two complexes per asymmetric unit, allowing collection of a synchrotron X-ray data set and refinement of the structure to a resolution of 1.8 Å (**Table S4**).

**Figure 2:**
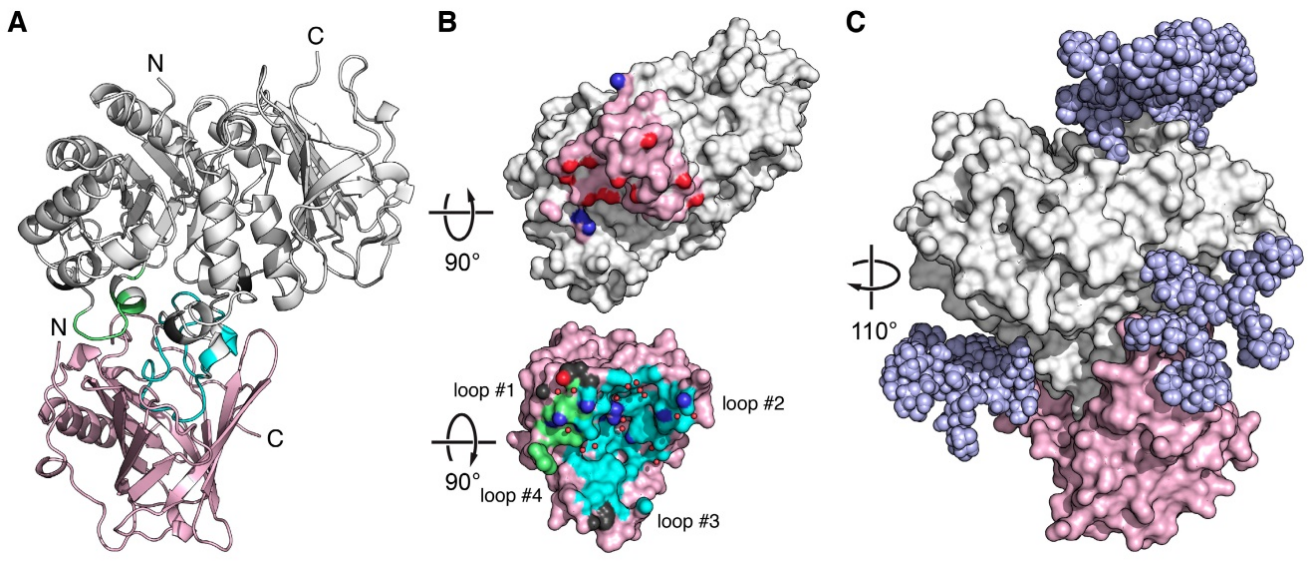
** X-ray structure of an Anticalin in complex with the human CD98hc ectodomain.** (A) Cartoon representation of the P3D11•hCD98hcED complex (P3D11 pink, hCD98hcED gray). The epitope loops L1 and L2 of hCD98hc are colored green and cyan, respectively. (B) Illustration of the interface after separating the two complex partners. Molecular contact surfaces are colored according to the opposite structure (cf. panel A) with hydrogen bond donors and acceptors highlighted blue and red, respectively, and hydrogen-bonded water molecules depicted as red spheres. (C) Surface representation of the P3D11•hCD98hcED complex including a model of the four complex N-glycans (light blue spheres) on the target protein.

hCD98hcED revealed a glycoside hydrolase fold, comprising a central TIM barrel core domain (residues 114-438) and a C-terminal β-sandwich domain (residues 439-529) as previously described 70. The engineered lipocalin binds to a membrane-distal part of hCD98hcED via the four variable loops #1-#4 at the open end of its β-barrel (**Figure 2A-B**). The β-barrel axis of P3D11 is tilted by ~45° with regard to the TIM barrel axis of hCD98hcED. The contact interface between both proteins involves a buried surface area (BSA) of 1473 Å^2^ (1424 Å^2^ on the side of the lipocalin and 1521 Å^2^ on the one of hCD98hcED) as well as 15 hydrogen bonds and 3 salt bridges (**Table S5**), resulting in the largest interface observed for Anticalin•protein complexes thus far 71. The interaction is favored by electrostatics as P3D11 is positively charged, which complements the predominantly negatively charged epitope on hCD98hcED, also reflecting the calculated p*I* values of 8.8 and 5.2 for P3D11 and hCD98hcED, respectively (**Figure S4A**).

The epitope of hCD98hcED essentially comprises two loops, L1 (residues 128-137) and L2 (374-404), which follow on the first and the eighth β-strand of the TIM barrel, respectively. Almost 75% of the contact interface is contributed by L2, which penetrates deeply into the binding pocket of the engineered lipocalin, while L1 contributes approximately 20% to the total BSA (**Table S5**). Further to the direct protein•protein contacts, 16 water molecules mediate hydrogen bonds at the interface. Based on the distribution of hydrogen bond donors, acceptors as well as water molecules, the P3D11 binding site towards hCD98hcED can be dissected into two areas, a rather polar region around the lipocalin loops #1 and #2 as well as a predominantly apolar region around loops #3 and #4 (**Figures 2B and S4B**).

Interestingly, binding of P3D11 causes an induced fit on the side of hCD98hcED. Superposition of the hCD98hcED Cα positions in the complex with P3D11 with those for hCD98hcED crystallized alone (PDB entry 2DH2) 70, excluding the epitope loops L1 and L2, revealed an RMSD value of 0.97 Å (for 375 equivalent Cα positions), whereas both loops L1 and L2 show a larger deviation in this context by on average 1.6 and 1.8 Å, respectively (**Figure S5A**). Likewise, P3D11 shows conformational differences among its loops #1-#4 compared with wtLcn2 (PDB code 1L6M); yet, these may also be caused by the many sequence changes (**Figure S5B**), as observed for other Anticalin structures before.

Although the selection of P3D11 was performed with the unglycosylated hCD98hcED, its affinity for the glycosylated hCD98hcEDg was indistinguishable (**Tables 1 and S3**). Modeling of the glycan moieties on the hCD98hcED shows that the Anticalin binds the protruding L1/L2 epitope in close proximity to two of the N-linked oligosaccharides without steric hindrance (**Figure 2C**). Interestingly, P3D11 does not show any crossreactivity with the murine antigen (mCD98hcED) as investigated via SPR measurements (data not shown), which can be explained by significant differences both in the amino acid sequence and in the glycosylation pattern between the two CD98hc orthologs. In particular, loop L2 of mCD98hcED is shorter by one residue, carries an additional N-glycosylation site and shows considerable sequence changes 72. Notably, this observation is in line with the properties of the clinical-stage mAb IGN523, which also recognizes loop L2 (residues 374-401) of hCD98hcED with picomolar affinity but shows no detectable affinity towards mCD98hc 22, 73.

### Directed evolution results in an Anticalin with improved affinity and stability

**Figure 3:**
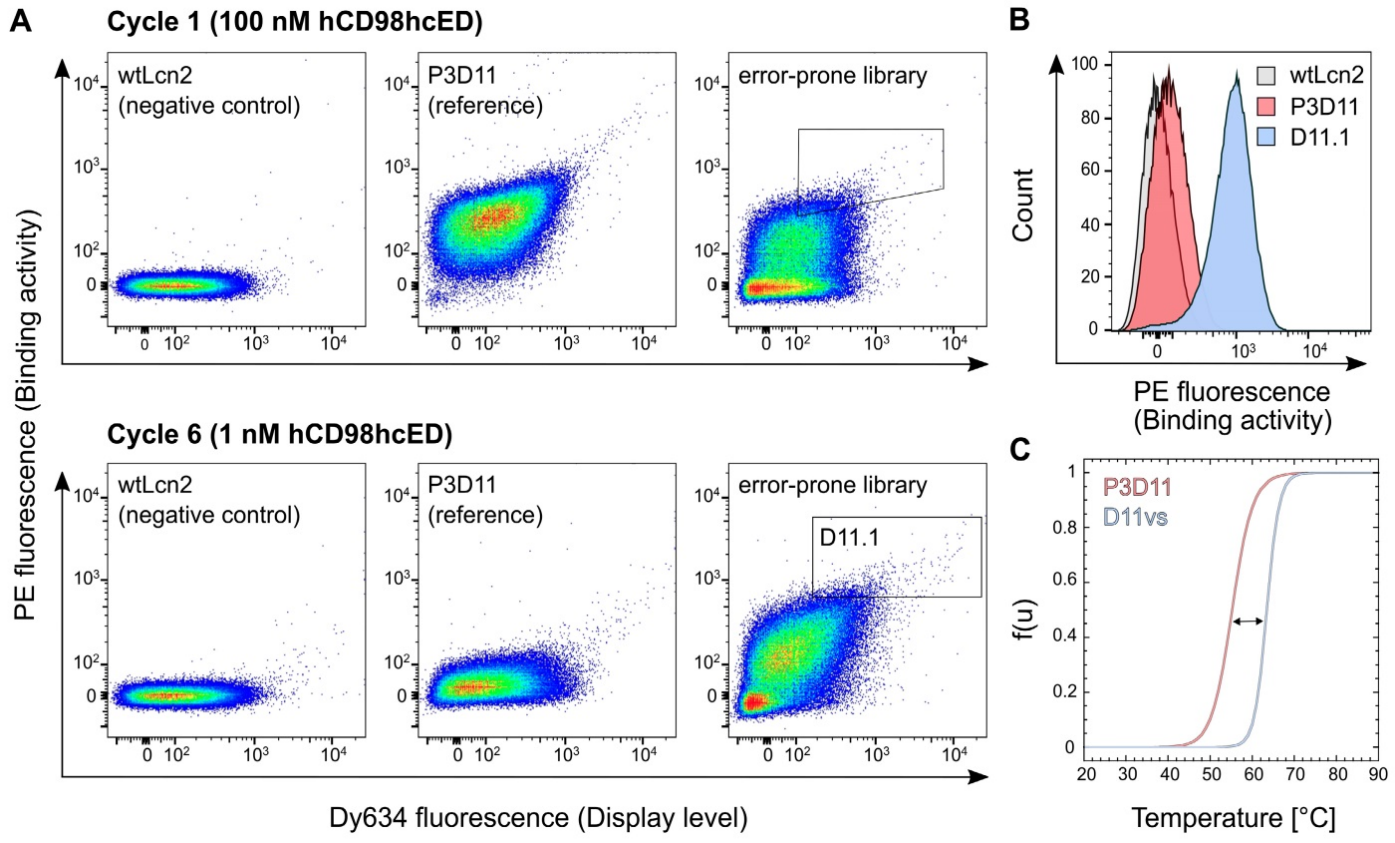
** Affinity and stability engineering of the lipocalin variant P3D11 via bacterial surface display.** (A) FACS analysis of *E. coli* cells presenting wtLcn2 (as negative control, left), the initial lipocalin variant P3D11 (as reference, middle) and the error-prone library (right), each incubated with 100 nM (cycle 1, upper panel) or 1 nM (cycle 6, lower panel) biotinylated hCD98hcED. (B) Single clone FACS analysis, using 1 nM biotinylated hCD98hcED, of P3D11 *versus* the second generation variant, D11.1, obtained after six selection cycles, in comparison with wtLcn2. (C) Thermal denaturation of P3D11 and its improved version D11vs measured by CD spectroscopy.

Despite its tight binding activity towards hCD98hcED, the lipocalin variant P3D11 suffered from low thermal stability, a caveat for further preclinical as well as clinical development (**Table 1**). To tackle this issue, stability engineering was performed via directed evolution using bacterial surface display (BSD) starting from an error-prone library with moderate amino acid mutation rate (approximately two amino acid exchanges on average; **Figure S6A**). To select variants of P3D11 with enhanced thermal stability, expression of the membrane-anchored protein in *E. coli* was induced at 37 °C (compared with 26 °C used in the initial selection) and only bacteria showing both a strong hCD98hcED binding signal and high cell surface display level were gated in the FACS experiment (**Figure 3A**).

After six enrichment cycles, the lipocalin library exhibited a pronounced average binding signal after incubation with 1 nM hCD98hcED, whereas almost no signal was detectable for the initial clone P3D11 if tested under the same conditions. Single clone analysis with 1 nM hCD98hcED verified strong binding of several new P3D11 variants (D11.1 shown as an example; see **Figures 3B and S6B**), whereas just very modest binding was detected for P3D11 under these conditions, and no signal for wtLcn2, as expected. Considering that P3D11 exhibits already quite a low K_D_ value of 150 pM for hCD98hcED, the increased binding signal is likely caused by the more efficient bacterial surface display due to improved protein folding and/or stability of its variants. Subsequent sequence analysis revealed that replacement of Gly81 and Phe71 in the P3D11 sequence by Val and Ser, respectively, were crucial in this regard (**Figure S6C**). Consequently, the variant D11vs, which carries both mutations, was produced as a soluble protein at preparative scale and characterized with respect to its thermal stability and target affinity.

CD thermal unfolding studies at physiological pH demonstrated a significant rise by 8.3 °C in the melting temperature (T_m_) for D11vs (T_m_ = 63.5 °C) compared to P3D11 (**Figure 3C and Table 1**). Importantly, also the cooperativity of the unfolding transition was much steeper, corresponding to a higher enthalpy of denaturation (∆H_m_) as well as extrapolated free energy of denaturation (∆G_U_) under standard conditions (25 °C). This is in line with our observation of a lower tendency of D11vs to aggregate during incubation at 37 °C compared to P3D11 (**Figure S6D**). Additionally, D11vs showed even higher affinity towards hCD98hcED, with a K_D_ value of 60 pM, due to both faster association and slower dissociation rate constants (**Figure S6E and Table 1**).

The enhanced stability and affinity of D11vs can be explained on the basis of the crystal structure solved for the P3D11•hCD98hcED complex: Gly81 was introduced during the initial selection campaign, where it replaced Arg81 of wtLcn2, thus creating space for tight interaction with the target (**Figure S6C**). However, due to the lack of a β-carbon, glycine causes a higher backbone conformational flexibility 74 which destabilizes the β-barrel. Accordingly, substitution by valine leads to enhanced protein stability while filling a hydrophobic cavity lined by the lipocalin residues Tyr68, Gly81, Phe83 and Leu94 (**Figure S7A**), of which also Tyr68 was introduced during the initial selection. In addition, Val81 increases the contact area with hCD98hcED, which likely contributes to the enhanced affinity (**Figure S7B**). On the other hand, replacement of Phe71 by Ser enables the formation of stabilizing hydrogen bonds to Asn174 and Lys50 within the Anticalin (**Figure S7C**), and the smaller side chain may enable its loop #2 to bend slightly away from the β-barrel axis, thus creating a better sterical situation for target binding.

### D11vs binds human CD98hc on various tumor cell lines

For *in vivo* studies in mice, a modified version of D11vs with moderately prolonged plasma half-life was designed using PASylation technology 75. To this end, a structurally disordered polypeptide comprising in total 200 proline, alanine and serine residues (PAS200) was genetically fused to the C-terminus of the engineered lipocalin (**Figure S8A**), thus increasing its hydrodynamic molecular volume, which leads to a retarded kidney filtration 50. In the context of *in vivo* imaging applications, previous investigations have shown that fine-tuning of the pharmacokinetics in this manner can boost protein tracer accumulation and tumor contrast 47. Furthermore, D11vs-PAS200 was equipped with an engineered Cys residue at the C-terminus of the PAS-tag to enable site-specific conjugation with chemical groups for *in vitro* and *in vivo* imaging as well as drug delivery (**Figure S8 and Table S2**).

**Figure 4:**
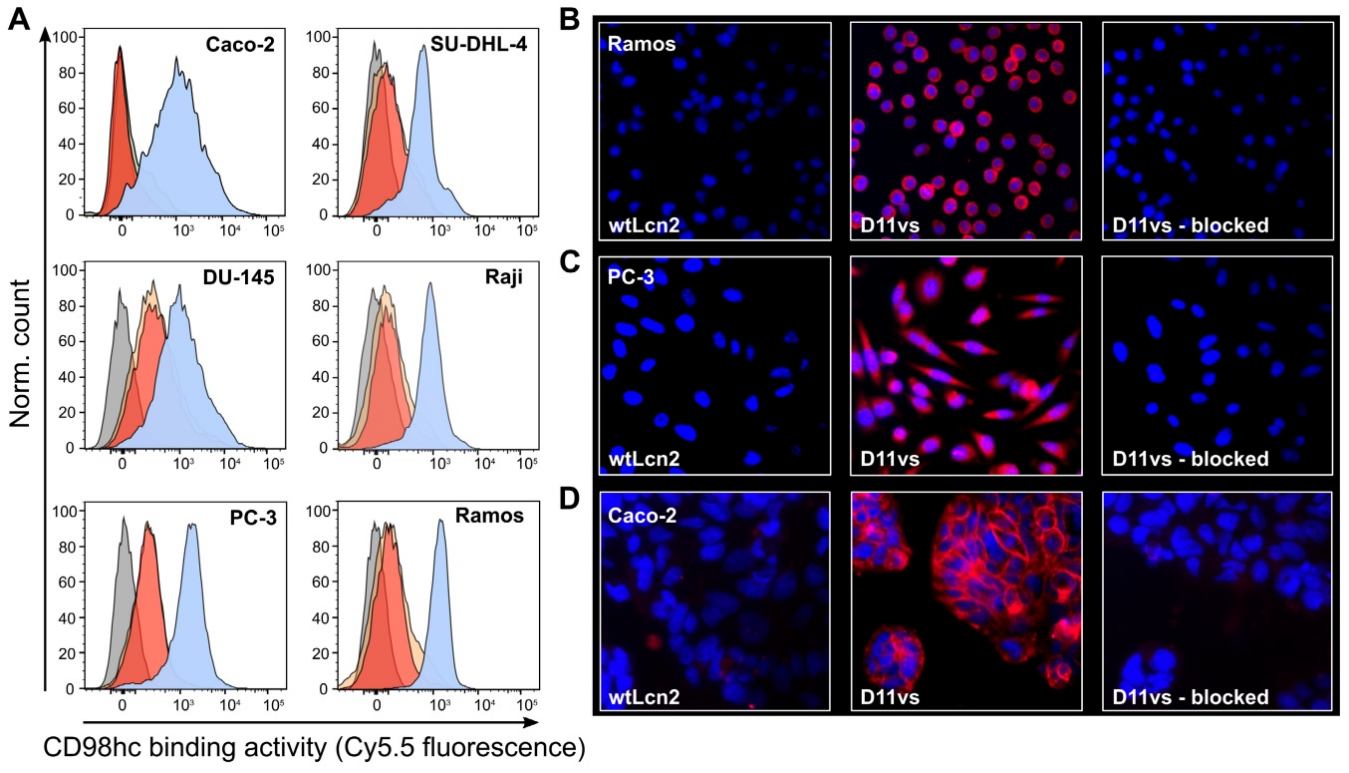
** Cytofluorometric and immunofluorescence microscopy of CD98hc expressing human cancer cell lines using a PASylated Anticalin.** (A) Flow cytometry analysis of cell lines from colorectal adenocarcinoma (Caco-2), prostate carcinoma (DU-145 and PC-3) and B-cell lymphoma (SU-DHL-4, Raji and Ramos) with Sulfo-Cy5.5-labeled PASylated D11vs (blue histograms). In a control experiment, a 10-fold molar concentration of either unlabeled D11vs-PAS200 or soluble hCD98hcEDg was co-applied for competition (red and yellow histograms, respectively). Untreated cell lines are shown in gray. (B, C, D) Immunofluorescent detection (red) of hCD98hc using Sulfo-Cy5.5-labeled PASylated D11vs shown for (B) Ramos, (C) PC-3 and (D) Caco-2 cells (middle). In control experiments, the cells were treated with Sulfo-Cy5.5-labeled wtLcn2 (left), or binding of Sulfo-Cy5.5-labeled PASylated D11vs was competed with a 10-fold molar concentration of unlabeled D11vs-PAS200 (right). Cell nuclei were counterstained with DAPI (blue).

The ability of D11vs-PAS200 to bind hCD98hc in a native cellular environment was investigated by cytofluorometry and immunofluorescence microscopy using human cancer cell lines of different origins that exhibit high expression of this tumor marker. Flow cytometric analysis of the B-cell lymphoma cell lines SU-DHL-4, Raji and Ramos, the prostate carcinoma cells DU-145 and PC-3 and the colorectal adenocarcinoma cell line Caco-2 using Sulfo-Cy5.5-labeled D11vs-PAS200 revealed strong binding to the membrane-associated target (**Figure 4A**). Competition experiments with excess unlabeled D11vs-PAS200 as well as soluble hCD98hcEDg resulted in signal suppression. The specificity of the Anticalin towards native hCD98hc was further confirmed by using a HEK293T knock-out cell line with an inactivated SLC3A2 gene (encoding the CD98hc membrane protein) 63, which also led to no FACS signal (**Figure S9**). Furthermore, immunofluorescence microscopy experiments with D11vs-PAS200-Cy5.5 revealed pronounced membrane and also cytoplasmic staining of Ramos, PC-3 and Caco-2 cancer cells (**Figure 4B-D**). Cell staining was effectively blocked when adding a 10-fold molar concentration of the unlabeled lipocalin variant, again demonstrating target specificity. As expected, no fluorescence staining for any of the tested cell lines was detected when using the Sulfo-Cy5.5-labeled PASylated wtLcn2.

### D11vs shows strong and specific signals in human tumor PET imaging of xenograft mouse models

**Figure 5:**
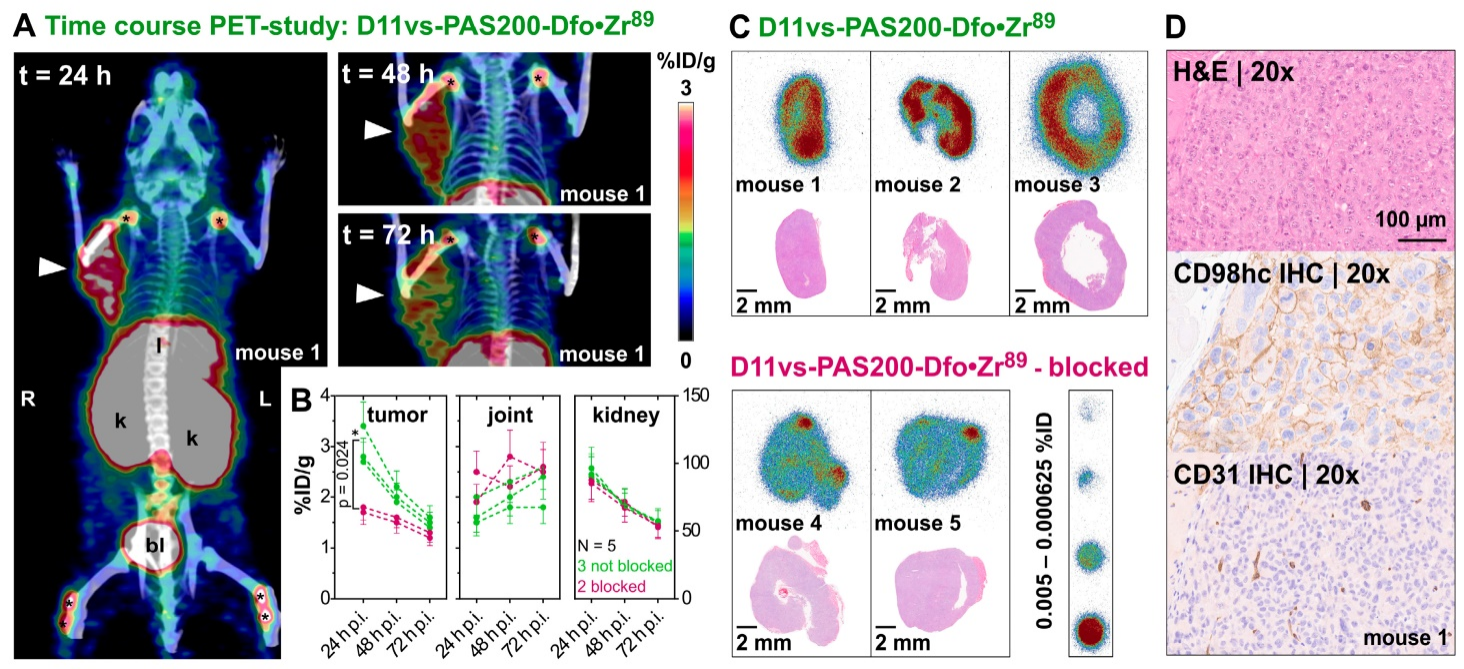
***In vivo* PET/CT imaging of a prostate carcinoma xenograft model.** (A) Mice (♀) bearing PC-3 xenografts were injected i.v. with 2.85 ± 0.15 MBq D11vs-PAS200-Dfo•^89^Zr and subjected to PET/CT imaging 24, 48 and 72 h post injection. Prominent signals were apparent in the xenograft tumor (arrowhead) as well as liver (l), kidneys (k), bladder (bl) and joints (*). (B) PET images were analyzed by threshold-based image segmentation (lower threshold at 50% of the hottest voxel), and activity trajectories were plotted. Data for mice with (green) and without (red) blocked target are shown. Epitope blocking was accomplished by i.v. injection of a 100-fold molar amount of the unlabeled PASylated Anticalin 2 h prior to application of the protein tracer. (C) Consecutive 10 µm cryosections were prepared from the tumor tissue followed by autoradiography and H&E staining. (D) The second half of each tumor was embedded in paraffin and sections were stained for CD98hc and CD31 (blood vessels), using appropriate antibodies, as well as with H&E.

The applicability of the PASylated Anticalin D11vs for the *in vivo* targeting of hCD98hc was evaluated by a PET/CT imaging study with the ^89^Zr-labeled protein in two murine xenograft tumor models. For this purpose, the human cancer cell lines Ramos and PC-3 were chosen, considering that CD98hc was described as a promising tumor marker for lymphoma 26 as well as other hematological cancers 15 and is known to be overexpressed in metastatic prostate cancer 25, 76. In a first study, ten female mice, five with a Ramos (**Figure S10**) and five with a PC-3 (**Figure 5**) xenograft, were injected with ~3 MBq of the radiolabeled protein, followed by PET/CT imaging at time points t = 24, 48 and 72 h p.i. To assess target specificity of the injected Anticalin in PC-3 xenografts, two of the five mice per group received a 100-fold molar amount of the unlabeled PASylated Anticalin 2 h before ^89^Zr-tracer injection. After 24 h, the best imaging contrast and high accumulation of 3.0 ± 0.3 %ID/g was observed for the PC-3 tumors, with decreasing tumor to background contrast after 48 and 72 h.

Threshold-based segmentation of the PET images (**Figure 5B**) showed a significant (p = 0.024) difference in radiotracer accumulation between tracer-injected mice (N=3) and those mice which were additionally injected with unlabeled Anticalin (N=2). Apart from the strong uptake by the tumors, elimination-related accumulation of radioactivity in liver, kidney and bladder was observed. Moreover, signals were seen in the joints, which can be explained by accumulation of liberated Zr(IV) due to the limited complex stability of the non-cyclic chelator Dfo 77. Notably, signals detected in joints and kidneys showed no significant difference in mice with or without blocked CD98hc epitopes, which confirms a tracer-independent uptake of radioactivity into these tissues (**Figure 5B**). Furthermore, the ^89^Zr-labeled D11vs-PAS200-Dfo tracer allowed good visualization of the Ramos xenografts 24 h p.i., yet with lower contrast as compared to the PC-3 tumors. Of note, the Ramos xenografts grew faster and more infiltrating than the PC3 xenografts, which made it difficult to accurately delineate these tumors on the PET images. Consequently, the PC-3 xenografts were chosen for further experiments.

After *in vivo* PET imaging, explanted PC-3 tumors were used for autoradiography of tissue cross sections. In this analysis, a homogeneous distribution of the radiotracer within the PC-3 tumor was detected (**Figure 5C**), with signals strongly reduced in tumors collected from the blocked mice. Concomitant immunohistochemical analysis of vessel distribution (via staining of CD31) as well as hCD98hc abundance, using cognate antibodies, revealed a well-vascularized tumor with high expression of hCD98hc for both PC-3 and Ramos xenografts (**Figures 5D and S11**).

**Figure 6:**
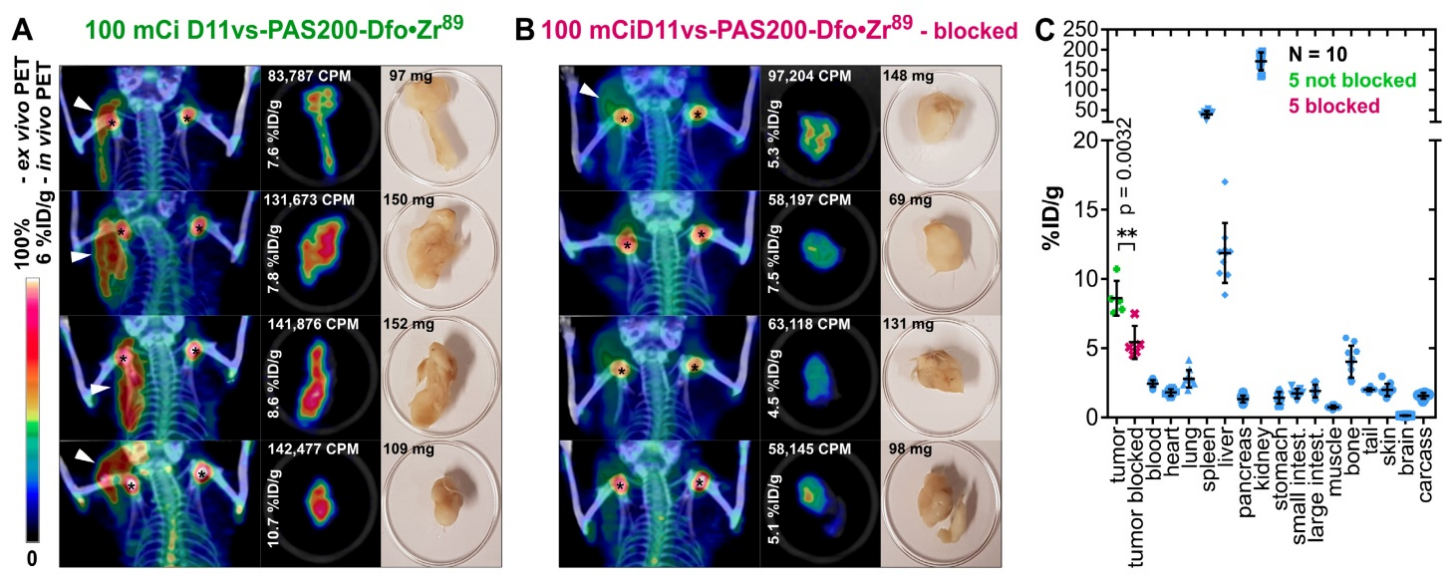
***In vivo* and *ex vivo* PET/CT imaging and biodistribution analysis.** (A) Mice (♂) bearing PC-3 tumors were injected with 3.96 ± 0.12 MBq D11vs-PAS200-Dfo•^89^Zr whereas mice in the blocked group (B) additionally received a 250-fold molar amount of unlabeled D11vs-PAS200 2 h prior to the protein tracer injection. For each mouse the *in vivo* PET/CT, *ex vivo* PET/CT and a photograph of the explanted tumor are depicted. (C) For biodistribution analysis, the mouse organs were dissected and their radioactivity and weight were quantified to allow calculation of the %ID/g values.

In order to confirm these imaging results and to obtain quantitative data from *ex vivo* biodistribution experiments at the optimal time point 24 h p.i., a second study using male CB17-SCID mice bearing PC-3 tumors was conducted (**Figure 6**). Two cohorts, each with five mice, were injected with either 3.96 ± 0.12 MBq ^89^Zr-labeled D11vs-PAS or with the protein tracer in combination with a 250-fold molar amount of the unlabeled PASylated Anticalin (at t = -2 h). After 24 h, PET/CT scans were performed, tumors were explanted, subjected to an *ex vivo* PET scan and, finally, a biodistribution analysis was conducted. PET/CT images of the mice without prior blocking of hCD98hc showed pronounced accumulation of radioactivity in the tumor, whereas the tumors were barely visible in the blocked mice, in line with the *ex vivo* PET/CT ROI segmentation analysis (p = 0.027). Finally, the biodistribution study revealed high accumulation of radioactivity (8.6 ± 1.1% ID/g) in the tumor (vs. 5.4 ± 1.1 %ID/g in the blocked cohort, p = 0.0032), with good tumor-to-blood and tumor-to-muscle ratios of 3.5 and 11.8, respectively. Radioactivity in the excretion-related organs matched the observations above, with additionally pronounced accumulation in the spleen. This can be explained by the formation of insoluble Zr-phosphate 78. In summary, the novel hCD98hc-specific PASylated Anticalin tracer showed high and specific tumor uptake in two xenograft models with excellent contrast, providing a proof of concept for exploiting CD98hc in non-invasive diagnostic imaging.

## Discussion

Due to its pathophysiological significance, CD98hc constitutes a highly attractive biomedical target for diagnostic as well as therapeutic applications in oncology. However, no CD98hc-specific reagents for *in vivo* imaging, and only few therapeutic drug candidates addressing this target, are available to date.

We have developed an Anticalin with exquisite specificity and high (picomolar) affinity towards the extracellular domain of hCD98hc. The initial Anticalin candidates were selected from a random library based on human Lcn2, an abundant siderophore-binding plasma protein. This previously designed library 51, which carries an optimally distributed set of randomized amino acid positions within the binding site of this protein scaffold, was successfully applied in previous studies to generate Anticalins against proteinaceous biomedical targets (ED-B, Hsp70, VEGFR-3 or PSMA) 51, 52, 68, 79, peptides (monomeric Aβ) 80 and hapten-type ligands (colchicine, Y(III)-DTPA, petrobactin) 53, 81. The lipocalin variants initially selected towards hCD98hcED from this naïve Lcn2 library exhibited K_D_ values already in the pico- to single-digit nanomolar range as well as stable monomeric behavior, which once again validates the utility of this protein scaffold and the library design.

Based on its favorable association and dissociation rate constants, with a remarkable complex dissociation half-life of ~6 h, P3D11 was chosen as the lead candidate for further characterization and optimization, eventually resulting in the Anticalin D11vs. X-ray structural analysis of the P3D11•hCD98hcED complex revealed deep penetration of the epitope loop L2, located in the membrane-distal part of the hCD98hc ectodomain, into the cup-shaped ligand pocket of the Anticalin, with a buried paratope surface area of 1424 Å^2^. Competition analysis for binding of the glycosylated hCD98hcg showed that all selected Anticalins in this study, including P3D11, recognize the same epitope region. P3D11 and P3A12 share a set of amino acid exchanges relative to wtLcn2, with 8 identical residues among the 20 randomized positions. Both Anticalins exhibit highly similar sequence stretches in loop #1, loop #2, as well as their adjoining β-strand segments A/B and C/D (**Figure S2A**), which contribute to the interface in the P3D11•hCD98hcED complex (**Table S5**).

While N-glycosylation of CD98hc has no influence on the target affinity of both P3D11 (or its improved version D11vs) and P3A12, binding of the third Anticalin candidate, P1E4, is significantly diminished (~100-fold). This behavior can be explained by minor variation in the mutual orientations between hCD98hcED and the different Anticalins, which may influence the sensitivity towards sterically demanding carbohydrate side chains in the neighborhood despite recognition of overlapping epitopes (**Figure 2C**). A similar kind of structural paratope plasticity was previously observed for a set of Anticalins directed against the extra-domain B of oncofetal fibronectin 71. Of note, the clinical-stage mAb IGN523 targets the same loop L2 (residues 374-401) of hCD98hcED, also with picomolar affinity 22, 73. Treatment of patients in a phase I clinical trial did not lead to severe adverse effects, suggesting that this epitope of CD98hc can be targeted safely.

In spite of its remarkable antigen affinity, suitable for *in vivo* tumor targeting, the Anticalin candidate P3D11 suffered from low thermal stability and pronounced aggregation after prolonged incubation at 37 °C. To overcome this obstacle, we employed directed evolution via moderate random mutagenesis in combination with high throughput sorting using a recently developed system for the bacterial surface display of Anticalins 54. By applying an elevated temperature during protein expression, the selection of variants showing improved protein folding and stability was enforced. With just two additional amino acid substitutions - Phe71Ser and Gly81Val (located in loop #2 and β-strand D, respectively) - the variant D11vs exhibited both improved affinity (approximately three-fold) as well as significantly increased melting temperature (by ~8 °C; cf. **Figure 3C and Table 1**), thus nicely illustrating the power of directed evolution for stability engineering of Anticalin proteins (**Figure S6C**).

Small animal PET/CT imaging and biodistribution analysis with the ^89^Zr-labeled D11vs radiotracer, whose plasma half-life was optimized using PASylation technology, showed high accumulation in PC-3 xenograft tumors with good imaging contrast and pronounced specificity, as blocking of CD98hc binding by co-application of unlabeled D11vs-PAS200 reduced tracer accumulation by ~40%. These findings were in line with *in vitro* cytofluorometry and immunofluorescence microscopy, where highly specific binding of D11vs on several CD98hc-positive human cancer cell lines, but not under conditions of a blocked CD98hc epitope, was evident. Tracer uptake in kidney, liver, spleen and joints of the xenograft mice was as expected due to the known *in vivo* release of ^89^Zr(IV) from the radiochelator 77, 82. Superior ^89^Zr-chelating reagents with higher *in vivo* stability, as for example fusarinine C, a cyclic siderophore from *Aspergillus fumigatus* 83, 84, may be an option for future experiments.

In conclusion, we have selected and engineered a human CD98hcED-specific Anticalin with high affinity and successfully applied it for CD98hc-targeted PET imaging. This novel radiopharmaceutical demonstrated high tumor uptake in prostate carcinoma as well as B-cell lymphoma xenograft mouse models with excellent tumor visualization. To our knowledge, this is the first *in vivo* imaging study of CD98hc overexpression on tumor cells described in the literature. Considering the clinical importance of elevated CD98hc expression in several human cancers, this Anticalin, charged with appropriate radionuclides or toxic payloads, constitutes a promising new tool for preclinical and, potentially, clinical applications in oncology.

## Supplementary Material

Supplementary methods, figures, and tables.

## Figures and Tables

**Table 1:** Affinities for hCD98hcED and thermal stabilities of engineered lipocalin variants

	Affinity				Stability		
Protein	K_D_ [nM]	k_on_ [M^-1^×s^-1^]	k_off_ [s^-1^]	τ_1/2_ [min]	T_m_ [°C]	∆H_m_ [kJ/mol]	∆G_U_ [kJ/mol]
P1E4	4.5 ± 0.026 ^a^	2.5×10^6^	1.1×10^-2^	1.1	68.8	812	104.0
P3A12	2.8 ± 0.024^ b^	6.2×10^4^	1.7×10^-4^	67.7	64.1	635	73.6
P3D11	0.15 ± 0.003^ b^	2.2×10^5^	3.2×10^-5^	359	55.2	368	33.8
D11vs	0.06 ± 0.007^ b^	4.5×10^5^	2.6×10^-5^	442	63.5	646	73.9

^a^ multi cycle kinetics ^b^ single cycle kinetics

## Abbreviations

BAP: biotin acceptor peptide
BSD: bacterial surface display
CD: circular dichroism
CT: computer tomography
CD98hc: Cluster of Differentiation 98 heavy chain
CD98lc: Cluster of Differentiation 98 light chain
FACBC: trans-1-amino-3-[^18^F]-fluorocyclobutanecarboxylic acid
FACS: fluorescence-activated cell sorting
FET: [^18^F]-fluoroethyl-L-tyrosine
m/hCD98hcED: mouse/human CD98hc extracellular domain
∆G^0^: Gibb's free energy of unfolding
∆H_m_: enthalpy of unfolding
%ID/g: percent injected dose per gram tissue
IMAC: immobilized metal ion affinity chromatography
K_D_: equilibrium dissociation constant
mAb: monoclonal antibody
PET: positron emission tomography
RMSD: root-mean-square deviation
ROI: region of interest
SPR: surface plasmon resonance
TLC: thin layer chromatography
T_m_: melting temperature
wtLcn2: wild type Lipocalin 2
