# Supplementary material for: Development of a high affinity Anticalin® directed against human CD98hc for theranostic applications
Source: Theranostics. 2020 Jan 12;10(5):2172–87. doi: 10.7150/thno.38968 (PMC7019167; doi:10.7150/thno.38968)
Supplement: Supplementary file 1 — Supplementary methods, figures, and tables. [file thnov10p2172s1.pdf]

SUPPORTING INFORMATION

Development of a high affinity Anticalin directed against human CD98hc for theranostic applications

Friedrich-Christian Deuschle[1], Volker Morath[2], André Schiefner[1], Corinna Brandt[1], Simone Ballke[3], Sybille Reder[2], Katja Steiger[3], Markus Schwaiger[2], Wolfgang Weber[2] and Arne Skerra[1*]

[1] Lehrstuhl für Biologische Chemie, Technische Universität München, 85354 Freising, Germany

[2] Department of Nuclear Medicine, Klinikum rechts der Isar, School of Medicine, Technical University of Munich, 81675 München, Germany

[3] Institute of Pathology, Klinikum rechts der Isar, School of Medicine, Technical University of Munich, 81675 München, Germany

*Corresponding author:

Prof. Dr. Arne Skerra, Lehrstuhl für Biologische Chemie, Technische Universität München, Emil-Erlenmeyer-Forum 5, 85354 Freising, Germany

Phone: +49 8161 71 4351; Fax: +49 8161 71 4352; e-mail: skerra@tum.de

SUPPORTING METHODS

Soluble production and purification of the monobiotinylated human and murine CD98hc ectodomains

The human and murine CD98hcED (UniProt ID P08195-2, residues Glu111–Ala529, and UniProt ID P10852-1, residues Glu105–Ala526, respectively) were produced in *E. coli* strain BL21 (New England Biolabs, Frankfurt/Main, Germany) using the expression plasmid pASK-IBA5(+)-BAP-m/hCD98hcED (Figure S1A) and in human embryonic kidney (HEK) 293E cells (MEXi-293E expression system; IBA Lifesciences, Göttingen, Germany) transiently transfected with pDSG-BM-His_6-BAP-m/hCD98hcEDg-Igk-BirA-*Strep*DEL (*Strep*DEL corresponds to a fusion between the *Strep*-tag II amino acid sequence, WSHPQFEK [1], and the endoplasmic reticulum retention signal sequence, KDEL [2]).

For expression in *E. coli*, BL21 was co-transformed with pBirAcm (Avidity, Aurora, CO) encoding biotin ligase, and heterologous gene expression was induced with 0.5 mg/L anhydrotetracycline (aTc) and 1 mM isopropyl-β-D-thiogalactopyranoside (IPTG) for 12 h at 26 °C in 2 L 2xYT medium. Bacteria were harvested by centrifugation, resuspended in anion-exchange chromatography (AEX) buffer (20 mM Tris/HCl pH 8.0, 1 mM EDTA) and disrupted with a PandaPLUS 2000 homogenizer (GEA Niro Soavi, Parma, Italy). The recombinant CD98hc ectodomain was purified from the whole cell extract by AEX using a MacroCap Q column (GE Healthcare, Munich, Germany) equilibrated with AEX buffer using a 3-step gradient (step 1: 50 mM; step 2: 190 mM; strep 3: 500 mM NaCl). Appropriate fractions from step 2 were pooled, dialyzed against 100 mM Tris/HCl pH 8.0, 50 mM NaCl, 1 mM EDTA, and applied to a 5 mL column carrying a streptavidin mutant which allows binding of biotinylated proteins and competitive elution via an excess of free biotin (unpublished). After elution using 5 mM biotin in the same buffer, preparative size-exclusion chromatography (SEC) was performed on a 24 mL Superdex 200 10/300 GL column (GE Healthcare) using PBS (4 mM KH_2PO_4, 16 mM Na_2HPO_4, 115 mM NaCl, pH 7.4) as running buffer.

Production of the m/hCD98hcEDg in MEXi-293E cells was essentially performed as recommended by the vendor of this expression system. Briefly, cells were cultivated in MEXi culture medium supplemented with 50 mg/l G-418 and 8 mM L-alanyl-L-glutamine at 37 °C under humidified 5% CO_2 atmosphere. 250 mL culture medium containing 5×10^6 cells/mL were transfected with 5 µg plasmid DNA per 1×10^6 cells, previously mixed at a 1:3 mass ratio with 25 kDa linear polyethyleneimine (PEI; Polysciences Europe, Eppelheim, Germany) in MEXi transfection medium. After 4 h incubation at 37 °C, 500 mL of MEXi cultivation medium was added and secretory expression was continued for 4 days. After sedimentation of the cells by centrifugation, the supernatant was dialyzed against immobilized metal ion affinity

chromatography (IMAC) buffer (20 mM Tris/HCl pH 8.0, 500 mM NaCl), and IMAC was performed on a Ni(II)-charged HisTrap HP column (GE Healthcare). Elution fractions obtained after applying a linear concentration gradient of 0–300 mM imidazole/HCl in IMAC buffer were pooled, followed by streptavidin affinity chromatography and preparative SEC as described above.

Analytical SEC and SDS-PAGE

Analytical SEC was performed on a 24 mL Superdex 200 10/300 GL column using PBS at a flow rate of 0.5 mL/min. For apparent molecular weight determination, the column was calibrated with the following standard proteins (Sigma-Aldrich, Munich, Germany): thyroglobulin (669 kDa), alcohol dehydrogenase (150 kDa), bovine serum albumin (66 kDa), carbonic anhydrase (29 kDa), cytochrome *c* (12.4 kDa) and aprotinin (6.5 kDa). The void volume of the column was determined using Blue Dextran (Sigma-Aldrich). Based on the elution volumes, the partition coefficients K_{av} were calculated and used to interpolate, via linear regression, the apparent molecular sizes of the analyzed proteins.

SDS-PAGE was performed using a high molarity Tris buffer system [3] with or without the addition of 2-mercaptoethanol in the sample buffer, followed either by staining with Coomassie brilliant blue or, for corresponding protein conjugates, by direct detection of Sulfo-Cy5.5 fluorescence using an Ettan DIGE fluorescence scanner (GE Healthcare) with an excitation wavelength of 635/30 nm and a 680/30 nm emission band-path filter.

Enzymatic cleavage of N-linked sugars from m/hCD98hcEDg

N-linked glycans were enzymatically removed from m/hCD98hcEDg produced in MEXi-293E cells using Peptide-N-Glycosidase F (PNGase F; New England Biolabs, Ipswich, MA). 5 µg protein in PBS were first denatured in the presence of 0.5% (w/v) sodium dodecyl sulfate (SDS) and 40 mM dithiothreitol (DTT) for 10 min at 100 °C. Subsequently, a 1/10 volume of 500 mM NaP_i pH 7.5 and of 10% (v/v) NP-40 as well as 250 units of PNGase F were added. After incubation for 1 h at 37 °C the sample was subjected to SDS-PAGE for comparison with the untreated ectodomain (Figure S1D).

Protein crystallization and structure determination

For protein crystallization, a variant of hCD98hcED with N-terminal *Strep*-tag II was produced in the *E. coli* strain BL21 as previously described [4] (Figure S1A). After incubation with the purified lipocalin variant P3D11 at 1:1 molar ratio for 1 h at 4 °C, the P3D11•hCD98hcED

complex was isolated via SEC on a Superdex 200 10/300 GL column (GE Healthcare) and directly eluted in crystallization buffer comprising 10 mM Hepes/NaOH pH 7.5, 100 mM NaCl, 0.02% (w/v) NaN_3. The complex was concentrated to 15.7 mg/mL using a 30 kDa MWCO Amicon ultracentrifugation filter (Merck Millipore, Burlington, MA) and subjected to crystallization by vapor diffusion in hanging drops at 20 °C. Diffraction quality crystals were obtained by mixing 1 µL of the P3D11•hCD98hcED solution with 1 µL of reservoir solution containing 18% (w/v) PEG3350 and 100 mM Na-malonate pH 4.75. Suitable crystals were transferred into cryoprotectant consisting of 19% (w/v) PEG3350, 100 mM Na-malonate pH 5.0 and 20% (v/v) ethylene glycol prior to flash cooling in liquid nitrogen.

X-ray diffraction data were collected at the Helmholtz-Zentrum Berlin, Germany, BESSY beamline 14.2 [5] and reduced with the XDS package [6] (Table S4). The crystal structure was solved by molecular replacement with Phaser [7] using the hCD98hcED (PDB entry 2DH2) [8] and the αED-B Anticalin N7A (PDB entry 4GH7) [9] as search models. Manual rebuilding and refinement were performed with Coot [10] and Refmac5 [11], respectively. Translation, libration and screw (TLS) groups were determined with TLSMD [12]. The asymmetric unit of space group C2 contained two P3D11•hCD98hcED complexes, of which the complex comprising the chain pairs A and B was used for the analysis described in this report due to the overall lower B-factors. Complex N-glycans were modeled on hCD98hcED as previously described [4].

Site-specific thiol labelling of PASylated lipocalin variants via maleimide chemistry

For site-specific labelling at the engineered C-terminal Cys side chain (Figure S8), maleimide-functionalized deferoxamine (Dfo; Macrocyclics, Plano, TX) or Sulfo-Cyanine 5.5 (Lumiprobe, Hannover, Germany) was used. To ensure the presence of a free thiol group for homogeneous C-terminal labelling, without cleaving the intramolecular disulfide bridge of the lipocalin variant, the purified protein was incubated with a 20-fold molar concentration of DTT for 1 h at 20 °C in PBS pH 7.4. Then, the buffer was exchanged against 50 mM NaP_i pH 5.5, 100 mM NaCl, 1 mM EDTA by gel filtration on a PD-10 column (GE Healthcare). After adjusting the pH to 7.4 using an appropriate volume of Na_3PO_4 and quantification of the protein concentration, a 5-fold molar concentration of the respective coupling reagent was added and incubated for 12 h at 4 °C. Residual reagents were removed using a Superdex 200 10/300 GL column equilibrated in PBS (pH 7.4). Successful 1:1 coupling with Dfo or Sulfo-Cy5.5 was verified using ESI-MS (Figure S8C-D) and the target affinity of each conjugate was measured by SPR spectroscopy (Table S2).

ESI mass spectrometry

Mass spectra of proteins were measured on a maXis mass spectrometer with an electrospray ionization (ESI) source (Bruker Daltonics, Bremen, Germany) in the positive ion mode. To measure the intact protein mass (under denaturing conditions), the purified protein was dialyzed against 10 mM ammonium acetate pH 6.6 followed by the addition of 50% (v/v) methanol and 0.1% (v/v) acetic acid and applied to the mass spectrometer via a syringe pump operated at 180 µL/h. The following conditions for the ion-transfer were used: 3400 V capillary voltage, 500 V endplate offset, 4 l/min dry gas at 200 °C temperature, 0.3 bar nebulizer pressure and 3 eV collision energy. Raw spectra were deconvoluted with the Bruker Compass Data Analysis Software using the MaxEnt algorithm [13] (cf. Table S2).

SUPPORTING REFERENCES

1. Voss S, Skerra A. Mutagenesis of a flexible loop in streptavidin leads to higher affinity for the *Strep*-tag II peptide and improved performance in recombinant protein purification. Protein Eng. 1997; 10: 975-82.
2. Denecke J, De Rycke R, Botterman J. Plant and mammalian sorting signals for protein retention in the endoplasmic reticulum contain a conserved epitope. EMBO J. 1992; 11: 2345-55.
3. Fling SP, Gregerson DS. Peptide and protein molecular weight determination by electrophoresis using a high-molarity tris buffer system without urea. Anal Biochem. 1986; 155: 83-8.
4. Deuschle FC, Schiefner A, Skerra A. Structural differences between the ectodomains of murine and human CD98hc. Proteins. 2019; 87: 693-8.
5. Mueller U, Darowski N, Fuchs MR, Förster R, Hellmig M, Paithankar KS, et al. Facilities for macromolecular crystallography at the Helmholtz-Zentrum Berlin. J Synchrotron Radiat. 2012; 19: 442-9.
6. Kabsch W. XDS. Acta Crystallogr D. 2010; 66: 125-32.
7. McCoy AJ, Grosse-Kunstleve RW, Adams PD, Winn MD, Storoni LC, Read RJ. Phaser crystallographic software. J Appl Crystallogr. 2007; 40: 658-74.
8. Fort J, de la Ballina LR, Burghardt HE, Ferrer-Costa C, Turnay J, Ferrer-Orta C, et al. The structure of human 4F2hc ectodomain provides a model for homodimerization and electrostatic interaction with plasma membrane. J Biol Chem. 2007; 282: 31444-52.
9. Gebauer M, Schiefner A, Matschiner G, Skerra A. Combinatorial design of an Anticalin directed against the extra-domain B for the specific targeting of oncofetal fibronectin. J Mol Biol. 2013; 425: 780-802.
10. Emsley P, Lohkamp B, Scott WG, Cowtan K. Features and development of Coot. Acta Crystallogr D 2010; 66: 486-501.
11. Murshudov GN, Skubak P, Lebedev AA, Pannu NS, Steiner RA, Nicholls RA, et al. REFMAC5 for the refinement of macromolecular crystal structures. Acta Crystallogr D 2011; 67: 355-67.
12. Painter J, Merritt EA. TLSMD web server for the generation of multi-group TLS models. J Appl Crystallogr. 2006; 39: 109-11.
13. Ferrige AG, Seddon MJ, Jarvis S, Skilling J, Aplin R. Maximum entropy deconvolution in electrospray mass spectrometry. Rapid Commun Mass Sp. 1991; 5: 374-7.
14. Davis IW, Leaver-Fay A, Chen VB, Block JN, Kapral GJ, Wang X, et al. MolProbity: all-atom contacts and structure validation for proteins and nucleic acids. Nucleic Acids Res. 2007; 35: W375-W83.

15. Baker NA, Sept D, Joseph S, Holst MJ, McCammon JA. Electrostatics of nanosystems: application to microtubules and the ribosome. Proc Natl Acad Sci U S A. 2001; 98: 10037-41.
16. Eichinger A, Nasreen A, Kim HJ, Skerra A. Structural insight into the dual ligand specificity and mode of high density lipoprotein association of apolipoprotein D. J Biol Chem. 2007; 282: 31068-75.
17. Goetz DH, Holmes MA, Borregaard N, Bluhm ME, Raymond KN, Strong RK. The neutrophil lipocalin NGAL is a bacteriostatic agent that interferes with siderophore-mediated iron acquisition. Mol Cell. 2002; 10: 1033-43.
18. Schiefner A, Gebauer M, Richter A, Skerra A. Anticalins reveal high plasticity in the mode of complex formation with a common tumor antigen. Structure. 2018; 26: 649-56.
19. Schönfeld D, Matschiner G, Chatwell L, Trentmann S, Gille H, Hülsmeyer M, et al. An engineered lipocalin specific for CTLA-4 reveals a combining site with structural and conformational features similar to antibodies. Proc Natl Acad Sci U S A. 2009; 106: 8198-203.
20. Skerra A. Lipocalins as a scaffold. Biochim Biophys Acta. 2000; 1482: 337-50.
21. Binder U, Matschiner G, Theobald I, Skerra A. High-throughput sorting of an Anticalin library via EspP-mediated functional display on the Escherichia coli cell surface. J Mol Biol. 2010; 400: 783-802.
22. Fuchs R, Stracke A, Holzmann V, Luschin-Ebengreuth G, Meier-Allard N, Ebner N, et al. Prazosin induced lysosomal tubulation interferes with cytokinesis and the endocytic sorting of the tumour antigen CD98hc. Biochim Biophys Acta Mol Cell Res. 2018; 1865: 1211-29.

SUPPORTING TABLES

Table S1. Size determination of the glycosylated and unglycosylated m/hCD98hcED via SEC

Protein	Theoretical mass [kDa]	Apparent size (SEC) [kDa]	Apparent size increase [%][a]
hCD98hcED	48.4	49.4	-
mCD98hcED	49.3	51.6	-
hCD98hcEDg	49.3	73.1	32.4
mCD98hcEDg	50.2	79.8	37.1

[a] caused by the N-linked glycosylation of h/mCD98hcED upon production in MEXi-293E cells *versus E. coli* BL21

Table S2. Protein mass determination of the Anticalins P1E4, P3A12, P3D11, D11vs and the PASylated D11vs versions via ESI-MS

Protein	Theoretical mass [Da]	Measured mass [Da]	Mass difference to uncoupled [Da]
P1E4	21656.6	21656.6 (Δ1.0)	-
P3A12	21340.2	21339.4 (Δ0.8)	-
P3D11	21100.8	21101.1 (Δ0.3)	-
D11vs	21082.8	21082.7 (Δ0.1)	-
D11vs-PAS200	37933.5	37933.6 (Δ0.1)	-
D11vs-PAS200-Cys	38036.7	n.d.	-
D11vs-PAS200-Dfo	38748.5 (Dfo: 711.8)	39749.4 (Δ0.9)	712.7
D11vs-PAS200-Cy5.5	39060.9 (S-Cy5.5: 1024.3)	39062.1 (Δ1.2)	1025.4

n.d. = not determined

Table S3. Affinities of lipocalin variants for hCD98hcEDg determined via SPR analysis

Lipocalin variant	K_D [a] [nM]	k_{on} [M^{-1}×s^{-1}]	k_{off} [s^{-1}]	$\tau_{1/2}$ [b] [min]
P1E4	162 ± 20.9	5.6×10^5	9.0×10^{-2}	<1
P3A12	1.9 ± 0.012	2.2×10^4	4.0×10^{-4}	29
P3D11	0.17 ± 0.004	3.0×10^5	5.0×10^{-5}	230
D11vs	0.05 ± 0.003	4.6×10^5	2.2×10^{-5}	523
D11vs-PAS200	0.44 ± 0.011	1.1×10^5	4.7×10^{-5}	245
D11vs-PAS200-Cys	0.32 ± 0.012	1.1×10^5	3.4×10^{-5}	338
D11vs-PAS200-Dfo	0.25 ± 0.003	3.1×10^5	7.7×10^{-5}	149
D11vs-PAS200-Cy5.5	0.31 ± 0.008	1.9×10^5	5.8×10^{-5}	198

[a] all measured via single cycle kinetics

[b] $\tau_{1/2}$ calculated from ln(2)/k_{off}

Table S4. X-ray data collection for the P3D11•hCD98hcED complex and refinement statistics[a]

Data collection	
Space group	C2
Unit cell parameters	a = 202.81 Å, b = 46.05 Å, c = 137.09 Å, $\alpha = \gamma = 90°$, $\beta = 106.64°$
Wavelength [Å]	0.9184
Resolution [Å]	30.0 - 1.80 (1.90 - 1.80)
Completeness [%]	99.8 (99.9)
Unique reflections	113253 (16844)
Multiplicity	6.8 (7.0)
Mean $I/\sigma(I)$	23.9 (2.3)
R_{meas} [%]	4.9 (93.4)
Wilson B-factor [Å]	37.4
Refinement	
Resolution [Å]	30.0 - 1.80 (1.85 - 1.80)
Reflections (working)	111018 (8149)
Reflections (test)[b]	2234 (187)
R_{cryst} [%]	18.4 (60.8)
R_{free} [%]	22.6 (67.0)
Protein molecules per au	4
Number of atoms: protein / solvent[c]	9356 / 909
B-values of atoms: protein / solvent [$Å^2$]	33.3 / 39.2
Ramachandran plot[d]: favored / outliers [%]	96.8 / 0.2
RMSD bonds [Å] / angles [°]	0.02 / 1.76

[a] Values in parentheses refer to the highest resolution shell.

[b] Test set corresponds to 2% of all reflections.

[c] Solvent refers to waters, ions, ordered buffer or cryoprotectant molecules

[d] Ramachandran statistics were calculated with MolProbity [14].

Table S5. Contacts between the P3D11 and hCD98hcED in the crystallized complex

	BSA [Å^2]	BSA mutated [Å^2]	HB / SB[a]
P3D11	1424	767	15 / 3
Loop #1	486	156	5 / 2
Loop #2	175	169	2 / -
Loop #3	90	1.0	1 / -
Loop #4	97	77	1 / -
β−barrel	576	364	6 / 1
hCD98hcED	1521	-	15 / 3
Loop L1	288	-	6 / 1
Loop L2	1111	-	6 / 1

[a] Hydrogen bonds (HB) / Salt bridges (SB), counting one SB per ion pair

SUPPORTING FIGURES

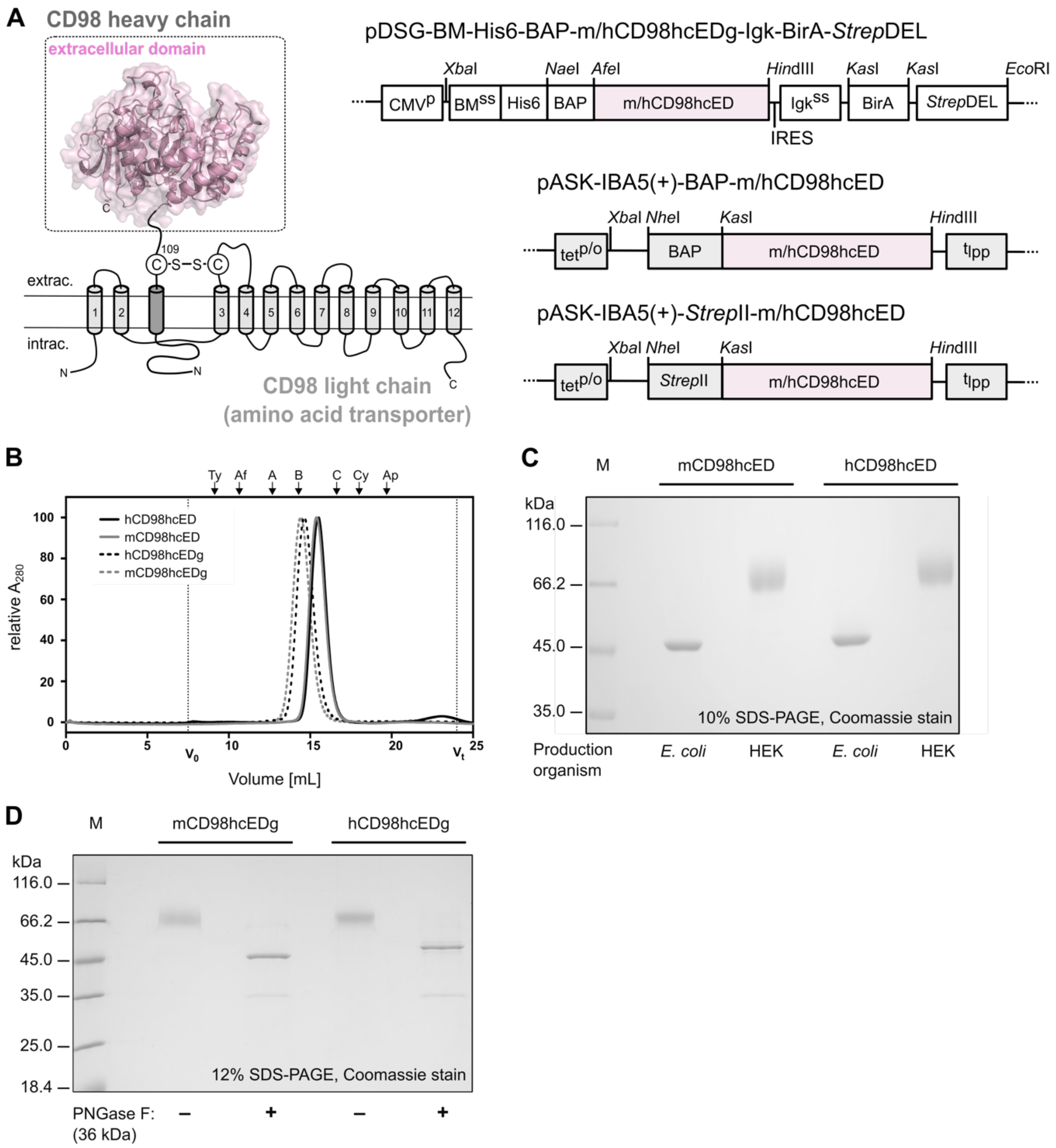

Figure S1. Biochemical characterization of the monobiotinylated human and murine CD98hc ectodomain produced in *E. coli* and MEXi-293E cells. (A) Schematic illustration of the covalently linked CD98hc/lc membrane receptor and plasmid constructs used for the soluble expression of the unglycosylated and glycosylated m/hCD98hc ectodomain. The extracellular domain of CD98hc used for Anticalin selection is highlighted by a rectangle. (B) Apparent size determination of the glycosylated and unglycosylated m/hCD98hcED using analytical SEC, verifying a considerable mass increase due to glycosylation (Table S1). (C) SDS-PAGE of m/hCD98hcEDg purified from MEXi-293E cells or *E. coli*, confirming glycosylation after production in the eukaryotic expression system as apparent from the much larger size. (D) Enzymatic cleavage of N-linked sugars from m/hCD98hcEDg by PNGase F (visible as a faint band at ~36 kDa) and comparison with the glycosylated ectodomain via SDS-PAGE.

A

```
       1        10        20        30        40        50        60        70        80        90
       .        .         .         .         .         .         .         .         .         .
Lcn2   QDSTSDLIPAPPLSKVPLQQNFQDNQFQGKWYVVGLAGNAILREDKDPQKMYATIYELKEDKSYNVTSVLFRKKKCDYWIRTFVPGCQPGEFTLGNIKS
Lib    ...........................h.......x...xx.......x..x...............x.x.xx...x.x.x.....s........x...
P1E4   ...........................h.......I...SM.......F..T...............R.R.DD...L.R.L.....s............
P3A12  ...........................h.......R...LG.......A..F...............Y.W.DL...K.S.H.....s........K...
P3D11  ...........................h.......R...TG.......G..F...............Y.W.GQ...M.S.G.....s............
                                  ====A=====------#1------===B===    ===C===--#2--=====D===      ==E=---#3

       100       110       120       130       140       150       160       170     178
       .         .         .         .         .         .         .         .       .
Lcn2   YPGLTSYLVRVVSTNYNQHAMVFFKKVSQNREYFKITLYGRTKELTSELKENFIRFSKSLGLPENHIVFPVPIDQCIDG
Lib    x..x..x..................x.x....x.x............................................
P1E4   R..W..W..................R.H....T.W............................................
P3A12  G..H..W..................W.G....N.A............................................
P3D11  A..R..W..................S.T....G.A............................................
      #3----===F====     ===G===---#4---===H===
```

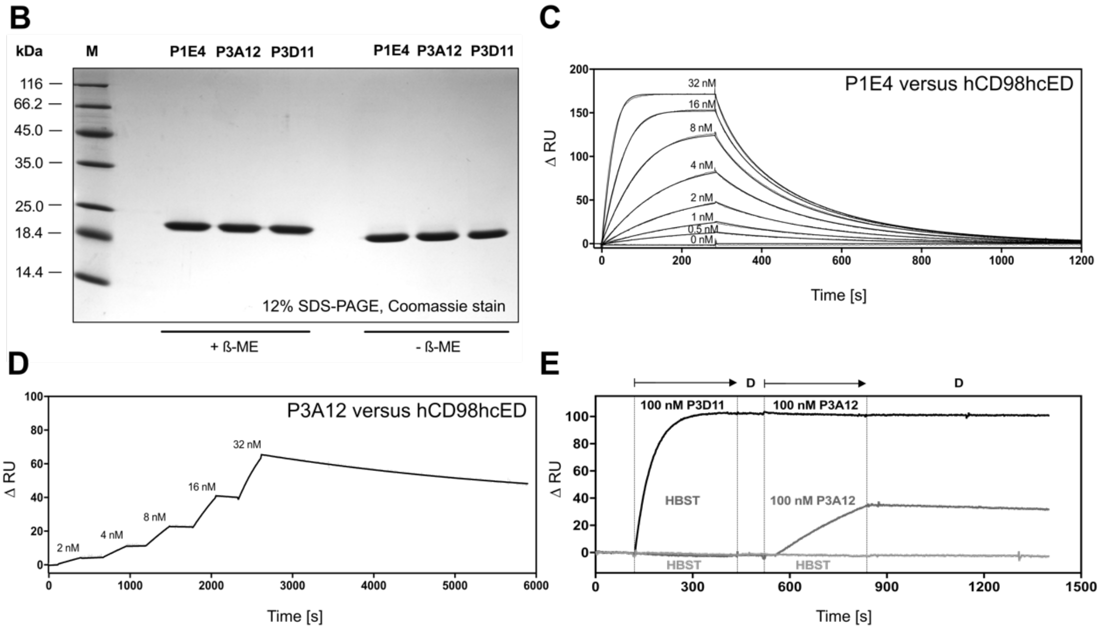

Figure S2. Sequence analysis, expression and functional characterization of the selected lipocalin variants P1E4, P3A12 and P3D11. (A) Amino acid sequence alignment compared to wtLcn2. The central randomized gene cassette flanked by a pair of *Bst*XI sites is underlined, β-stands and structurally hypervariable loops are labeled with letters A–H and numbers #1–#4, respectively. (B) Coomassie-stained SDS-PAGE of the recombinant lipocalin variants after production in *E. coli*. The increased electrophoretic mobility under non-reducing conditions indicates formation of the single structural disulfide bridge in the Lcn2 scaffold. (C, D) Biomolecular interaction analysis between the immobilized hCD98hc ectodomain produced in *E. coli* (ΔRU = 225) and the selected lipocalin variants P3A12 and P1E4 via SPR measurements using multi-cycle (C) and single-cycle (D) procedures. (E) Competitive binding analysis between the lipocalin variants P3D11 and P3A12 by SPR measurement. Binding sites on hCD98hcEDg were saturated with an injection of 100 nM P3D11 followed by injection of 100 nM P3A12, which did not cause an additive RU signal (black line). In contrast, injection of buffer followed by 100 nM P3A12, without prior blocking of the hCD98hcED epitope with P3D11, led to a binding signal for P3A12 (dark gray line). Mock buffer injections are shown as negative control (light gray line).

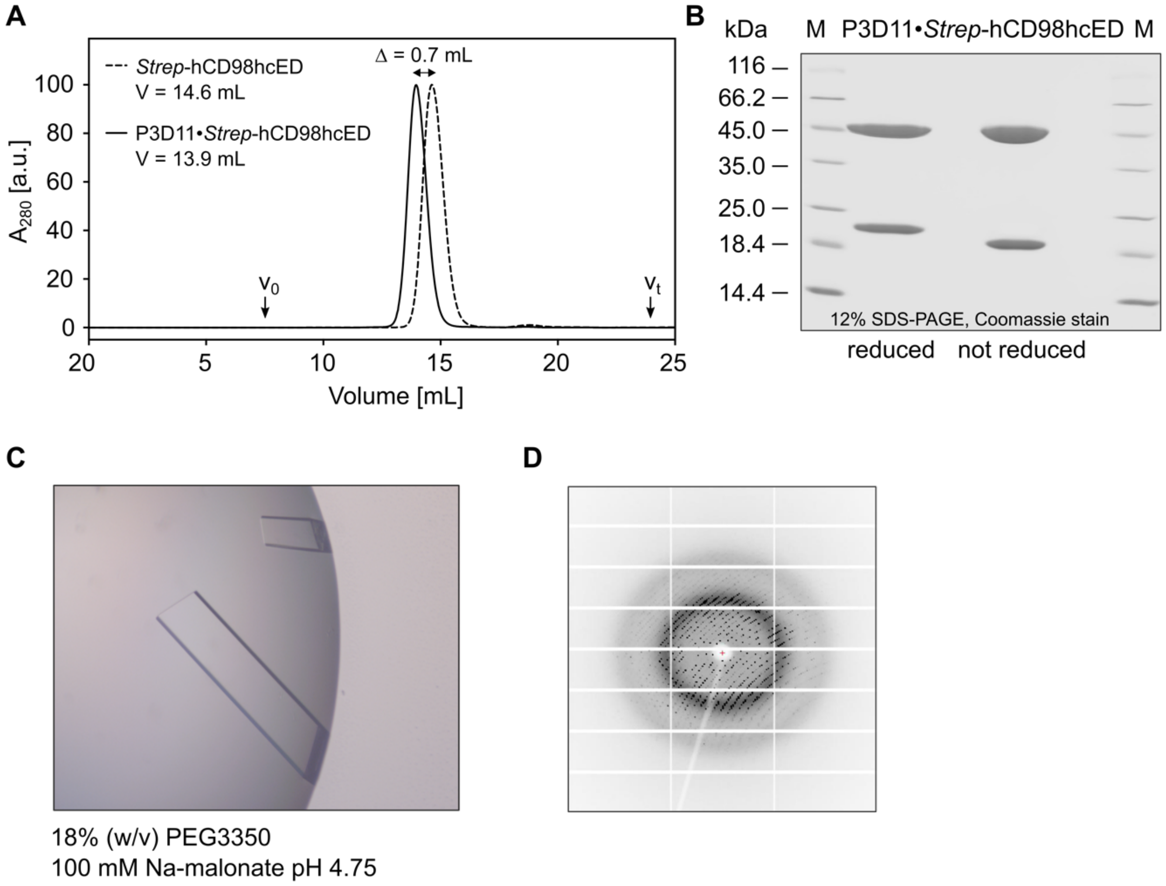

Figure S3. Crystallization of the P3D11•hCD98hcED complex. (A) SEC purification of the 1:1 complex prepared by mixing both recombinant proteins. (B) Analytical SDS-PAGE of the isolated P3D11•hCD98hcED complex with and without the addition of 2-mercaptoethanol. (C) Diffraction quality crystals obtained by vapor diffusion in hanging drops at 20 °C in 18% (w/v) PEG3350 and 100 mM Na-malonate pH 4.75. (D) X-ray diffraction pattern of the large crystal in (C) collected at a wavelength of 0.9184 Å using a Pilatus 2M detector.

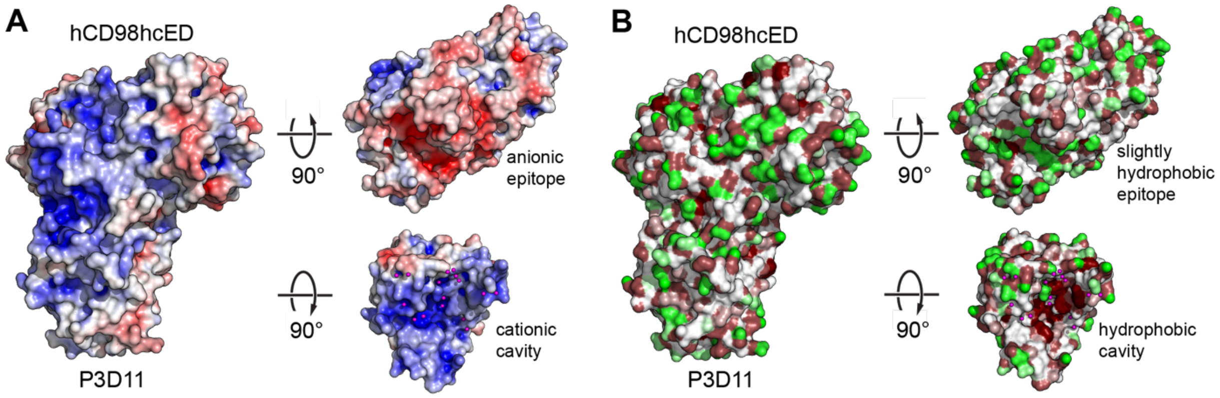

Figure S4. Electrostatic and hydrophobic properties of the P3D11•hCD98hcED complex interface. (A) Electrostatic surface potentials were calculated with APBS [15] and colored from -10 $k_B T/e$ (red) to +10 $k_B T/e$ (blue). (B) Surface hydrophobicity values are shown from brown (hydrophobic) over white (neutral/peptide backbone) to green (polar) [16].

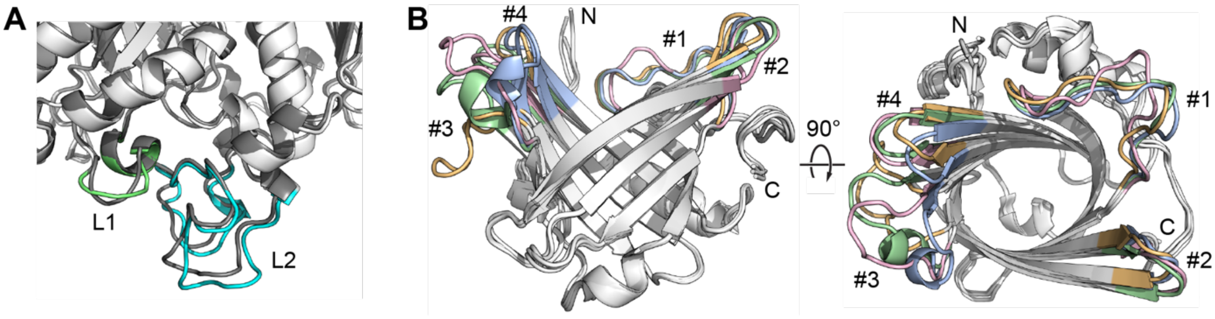

Figure S5. Conformational variations in the components of the P3D11•hCD98hcED complex. (A) Conformational changes of the hCD98hcED loops L1 (green) and L2 (cyan) upon complex formation with P3D11 in comparison with the unbound hCD98hcED (dark gray; PDB code 2DH2). (B) Structural superposition of P3D11 with wtLcn2 (PDB code 1L6M) [17], the fibronectin-specific Anticalin N7E (PDB code 5N47) [18] and the CTLA4-specific Anticalin PRS#003 (PDB code 3BX7) [19] using 58 Cα positions that are structurally conserved among the lipocalins [20]. The structurally variable loops are colored pink, blue, yellow and green for P3D11, wtLcn2, N7E and PRS#003, respectively.

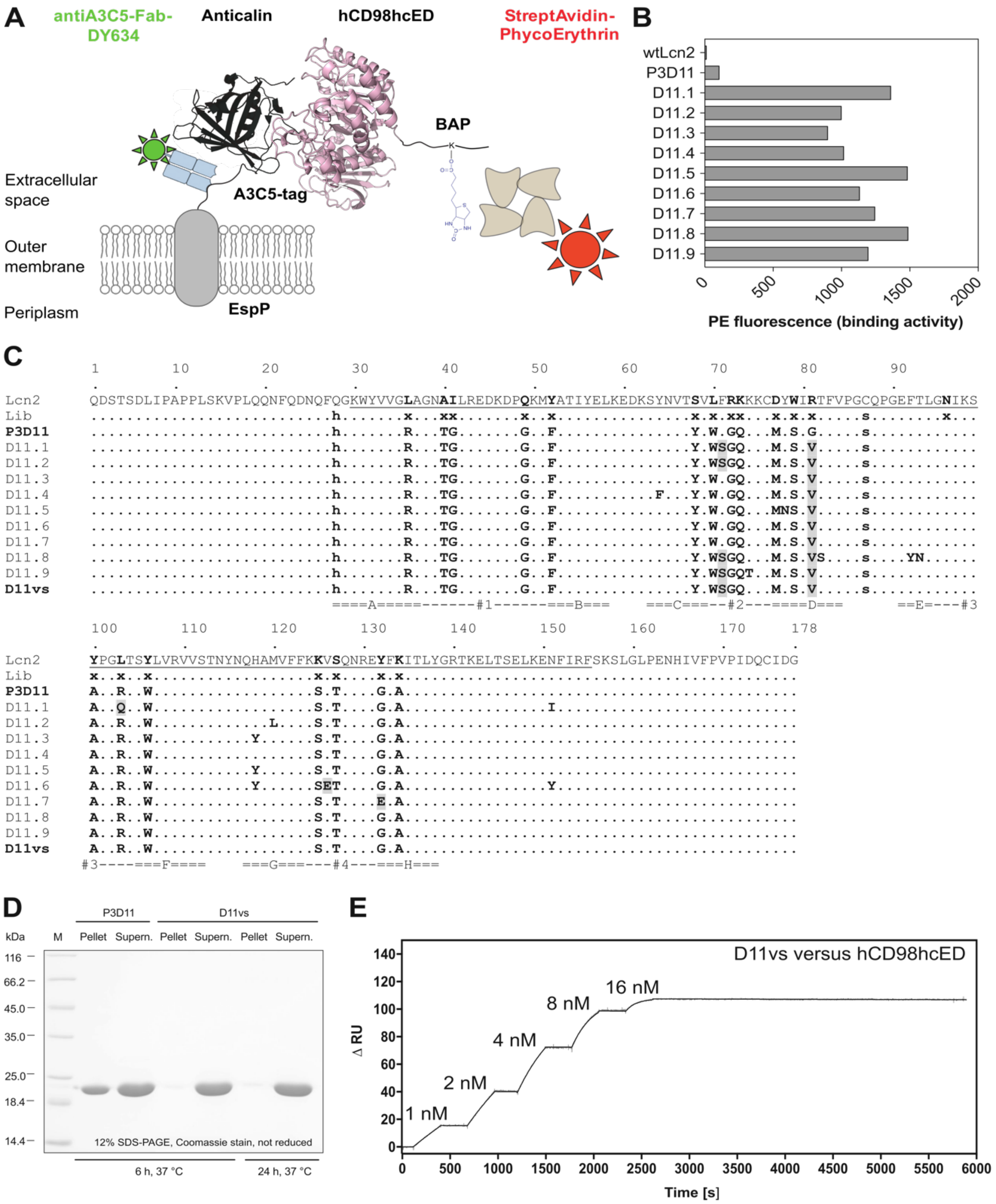
A
antiA3C5-Fab-DY634
Anticalin
hCD98hcED
StreptAvidin-PhycoErythrin
BAP
Extracellular space
A3C5-tag
Outer membrane
Periplasm
EspP
B
wtLcn2
P3D11
D11.1
D11.2
D11.3
D11.4
D11.5
D11.6
D11.7
D11.8
D11.9
0
500
1000
1500
2000
PE fluorescence (binding activity)
C
Lcn2
Lib
P3D11
D11.1
D11.2
D11.3
D11.4
D11.5
D11.6
D11.7
D11.8
D11.9
D11vs
QDSTSDLIPAPPLSKVPLQQNFQDNQFQGKWYVVGLAGNAILREDKDPQKMYATIYELKEDKSYNVTSVLFRKKKCDYWIRTFVPGCQPGEFTLGNIKS
YPGLTSYLVRVVSTNYNQHAMVFFKKVSQNREYFKITLYGRTKELTSELKENFIRFSKSLGLPENHIVFPVPIDQCIDG
D
P3D11
D11vs
kDa
M
Pellet
Supern.
116
66.2
45.0
35.0
25.0
18.4
14.4
12% SDS-PAGE, Coomassie stain, not reduced
6 h, 37 °C
24 h, 37 °C
E
D11vs versus hCD98hcED
1 nM
2 nM
4 nM
8 nM
16 nM
Δ RU
Time [s]

Figure S6. Engineering of the lipocalin variant P3D11 via error prone PCR and bacterial surface display. (A) Schematic depiction of the Lcn2 scaffold presented on the outer membrane of *E. coli* via fusion with the engineered β-domain of the auto-transporter protein EspP [21]. (B) Single clone FACS analysis in the presence of 1 nM biotinylated hCD98hcED after six BSD selection cycles. The mean intensity of PE fluorescence is shown for the individual improved lipocalin variants in comparison with the starting variant P3D11 and with wtLcn2. (C) Amino acid sequence alignment of the improved lipocalin variants derived from P3D11. The variant D11vs was constructed based on this sequence information. (D) Stability testing of the lipocalin variant P3D11 and its engineered version D11vs via incubation at 37 °C for 6 or 24 h followed by SDS-PAGE analysis, indicating aggregate formation (i.e., protein pellet) in the case of P3D11. (E) SPR real-time binding study of the improved variant D11vs toward hCD98hcED using single cycle kinetics. The deduced kinetic constants are listed in Table 1.

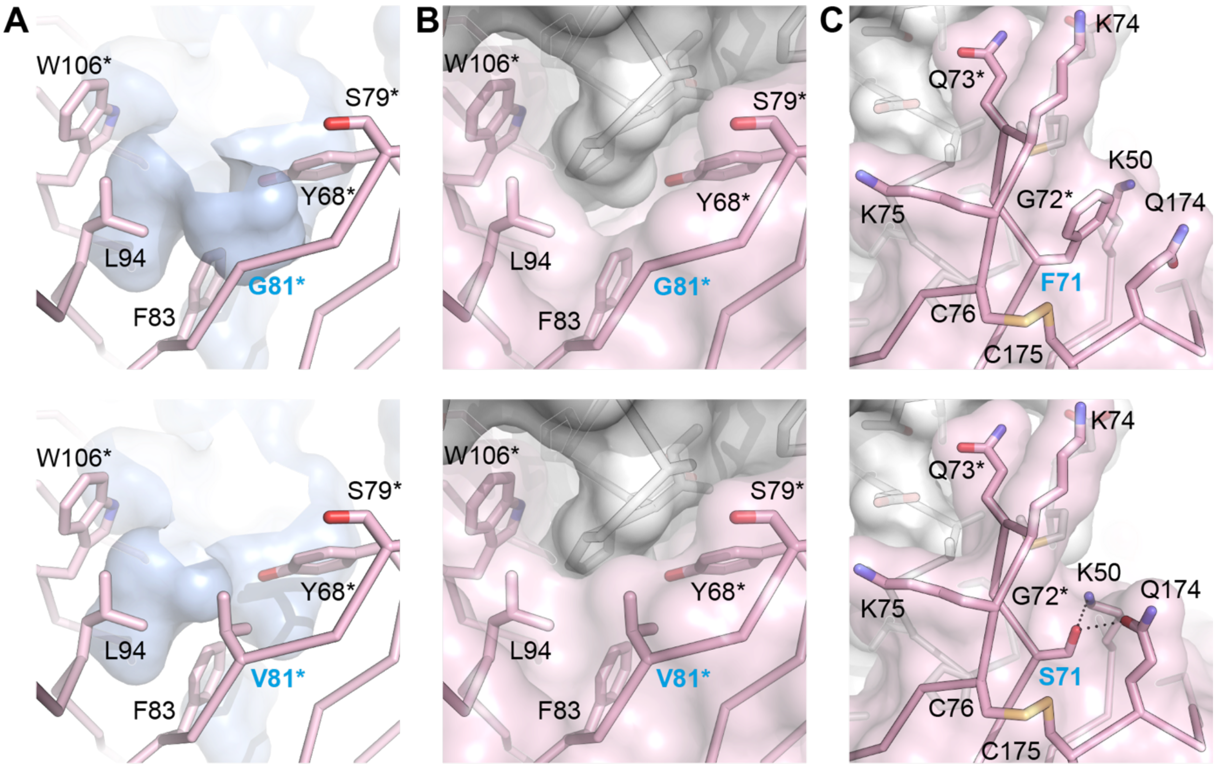

Figure S7. Structural context of side chain substitutions that increase stability and/or target affinity of P3D11. Residues that differ from the Lcn2 scaffold are labeled with asterisks. (A) Hydrophobic cavity (light blue) at the open end of the β-barrel of the lipocalin lined by residues Tyr68, Gly81, Phe83 and Leu94 (top). Substitution of Gly at position 81 by Val partially fills this cavity (bottom). (B) In addition, Val81 improves the contact interface with hCD98hc. (C) Substitution of Phe71 by Ser disrupts the π-stacking between Phe71 and Gln174 but permits hydrogen bond formation with Gln174 and Lys50.

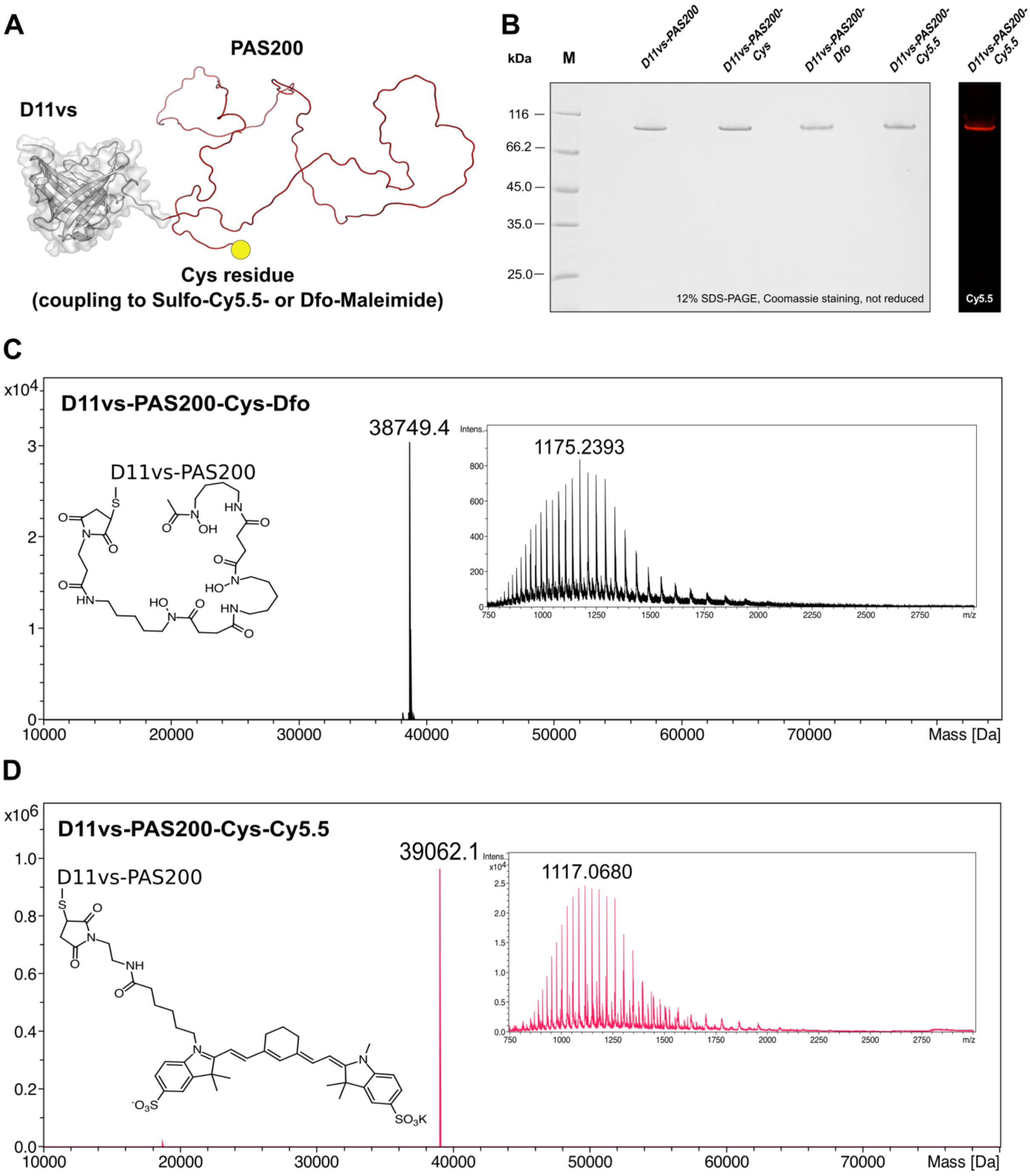

Figure S8. Characterization of PASylated D11vs and its conjugates. (A) Molecular model of the Anticalin D11vs (gray) with a C-terminally attached PAS200 polypeptide (red), including an engineered Cys residue (yellow) at the end. (B) Non-reducing SDS-PAGE of the PASylated D11vs variants used for *in vitro* and *in vivo* studies. For the D11vs-PAS200-Cy5.5 conjugate (rightmost lane), fluorescence was also detected on a fluorescence scanner. (C and D) ESI-MS (deconvoluted, raw data are shown as inset) for D11vs-PAS200-Cys after coupling to Sulfo-Cy5.5 (C) or Dfo (D) using maleimide chemistry, thus confirming homogeneous site-specific labelling (Table S2).

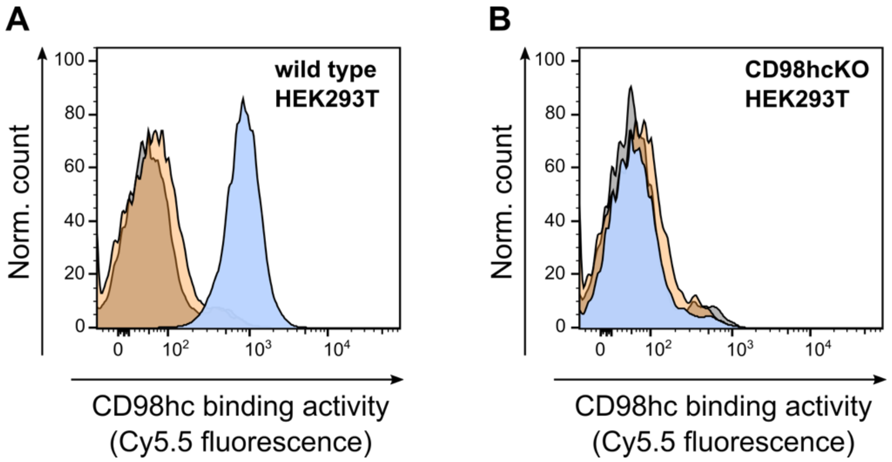

Figure S9. Cytofluorometric staining of CD98hc expressing HEK293T cells in comparison with a derivative CD98hc knock-out (KO) cell line using a PASylated Anticalin. (A) Flow cytometry of wild type HEK293T cells with Sulfo-Cy5.5-labeled PASylated D11vs (blue histogram). In control experiments, a 10-fold molar concentration of soluble hCD98hcEDg was co-applied for competition (orange histogram) or the cells were treated with Sulfo-Cy5.5-labeled PASylated wtLcn2 (gray histogram). (B) To test the specificity of the Sulfo-Cy5.5-labeled PASylated D11vs, a HEK293T cell line with a CRISPR/Cas9-mediated SLC3A2 gene knock-out was investigated using the same reagents [22].

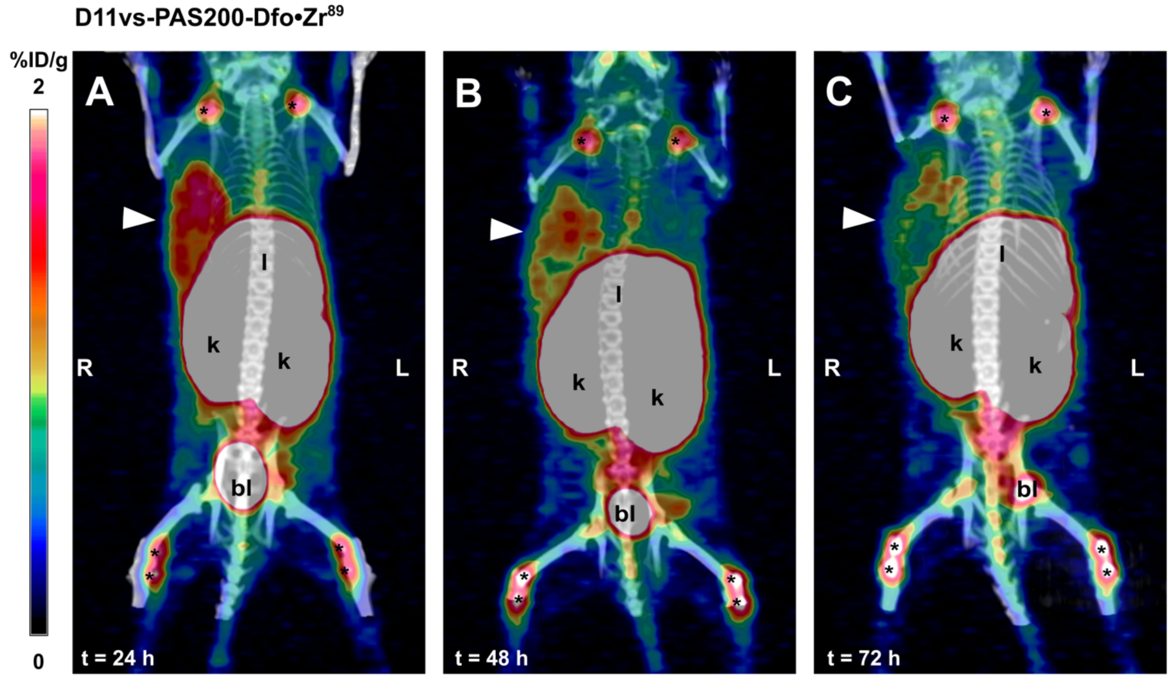

Figure S10. ***In vivo*** **^{89}Zr PET/CT imaging study with PASylated D11vs in a Non-Hodgkin's lymphoma xenograft model.** Mice (♀) bearing a Ramos xenograft were injected i.v. with 2.9 MBq D11vs-PAS200-Dfo•Zr^{89} followed by PET/CT imaging at time points (A) 24, (B) 48 and (C) 72 h p.i. Signals were detected in the xenograft tumor (arrowheads), liver (l), kidneys (k), bladder (bl) and the joints (*).

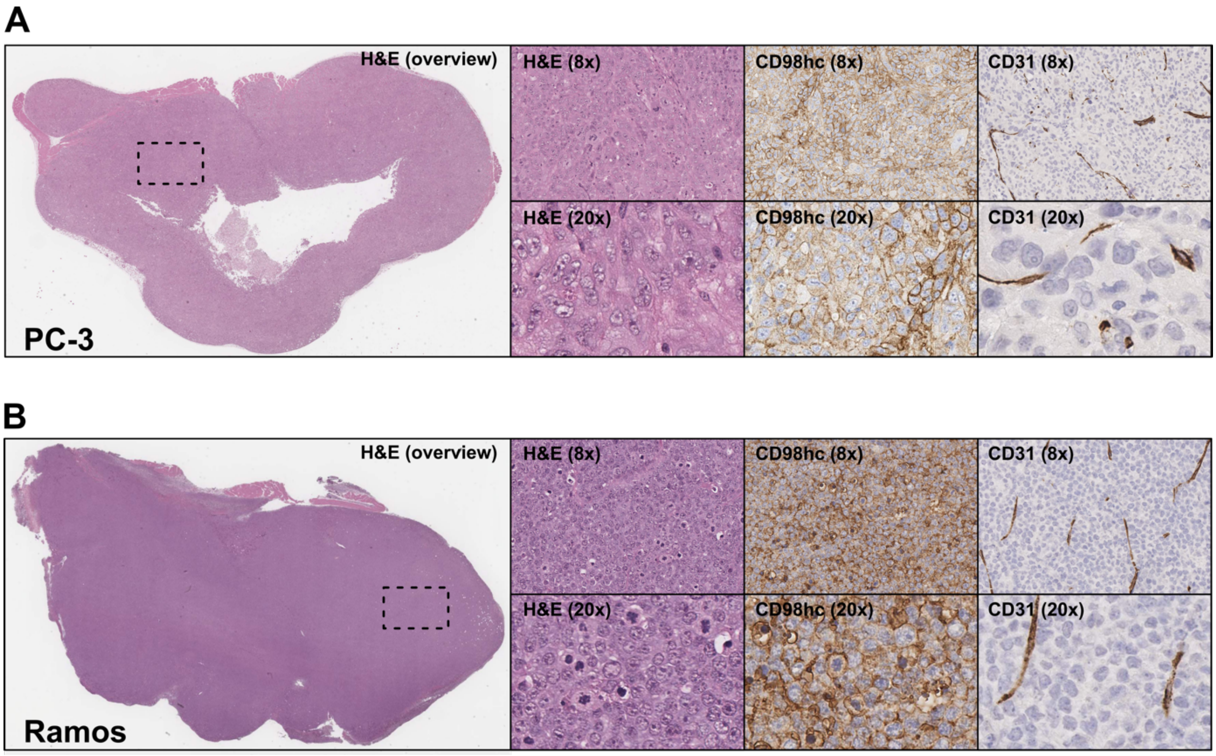

Figure S11. Immunohistochemical characterization of PC-3 and Ramos xenograft tumor sections. Tissue sections of PC-3 (A) and Ramos (B) tumors were stained with H&E as well as with antibodies against CD31 (blood vessels) and CD98hc (magnification is indicated). PC-3 tumor xenografts appeared as solid growing carcinomas with central necrosis. Tumor cells showed numerous mitoses and pale, vacuolated nuclei with multiple nucleoli. The tumors were well-vascularized and showed moderate to strong membrane expression of CD98hc in 80% of the cells, with slight intratumoral heterogeneity. Ramos xenografts were also well-vascularized, consisting of large blastoid cells (lymphoblasts) with high mitotic activity. CD98hc expression was evident in 100% of these cells as revealed by moderate to severe membrane staining.
